# Predicting genome-wide DNA methylation using methylation marks, genomic position, and DNA regulatory elements

**DOI:** 10.1186/s13059-015-0581-9

**Published:** 2015-01-24

**Authors:** Weiwei Zhang, Tim D Spector, Panos Deloukas, Jordana T Bell, Barbara E Engelhardt

**Affiliations:** Department of Molecular Genetics and Microbiology, Duke University, Durham, NC USA; Department of Twin Research and Genetic Epidemiology, King’s College London, London, UK; William Harvey Research Institute, Barts and The London School of Medicine and Dentistry, Queen Mary University of London, London, UK; Princess Al-Jawhara Al-Brahim Centre of Excellence in Research of Hereditary Disorders (PACER-HD), King Abdulaziz University, Jeddah, 21589 Saudi Arabia; Department of Computer Science, Princeton University, Princeton, NJ USA

## Abstract

**Background:**

Recent assays for individual-specific genome-wide DNA methylation profiles have enabled epigenome-wide association studies to identify specific CpG sites associated with a phenotype. Computational prediction of CpG site-specific methylation levels is critical to enable genome-wide analyses, but current approaches tackle average methylation within a locus and are often limited to specific genomic regions.

**Results:**

We characterize genome-wide DNA methylation patterns, and show that correlation among CpG sites decays rapidly, making predictions solely based on neighboring sites challenging. We built a random forest classifier to predict methylation levels at CpG site resolution using features including neighboring CpG site methylation levels and genomic distance, co-localization with coding regions, CpG islands (CGIs), and regulatory elements from the ENCODE project. Our approach achieves 92*%* prediction accuracy of genome-wide methylation levels at single-CpG-site precision. The accuracy increases to 98*%* when restricted to CpG sites within CGIs and is robust across platform and cell-type heterogeneity. Our classifier outperforms other types of classifiers and identifies features that contribute to prediction accuracy: neighboring CpG site methylation, CGIs, co-localized DNase I hypersensitive sites, transcription factor binding sites, and histone modifications were found to be most predictive of methylation levels.

**Conclusions:**

Our observations of DNA methylation patterns led us to develop a classifier to predict DNA methylation levels at CpG site resolution with high accuracy. Furthermore, our method identified genomic features that interact with DNA methylation, suggesting mechanisms involved in DNA methylation modification and regulation, and linking diverse epigenetic processes.

**Electronic supplementary material:**

The online version of this article (doi:10.1186/s13059-015-0581-9) contains supplementary material, which is available to authorized users.

## Background

Epigenetics is the study of non-genetic cellular processes that may be inherited, are stable through cell division, and may change in response to external and internal cellular stimuli. Epigenetic markers may change within an individual over time and have been shown to exhibit cell-type specificity [1-3]. Epigenetics has been shown to play a critical role in cell differentiation, development, and tumorigenesis [4,5]. DNA methylation is probably the best studied epigenetic modification of DNA, but our understanding of DNA methylation is still in its infancy. In vertebrates, DNA methylation occurs when a methyl group is added to the fifth carbon of the cytosine residue, mainly in the context of neighboring cytosine and guanine nucleotides in the genome (5-CG-3 dinucleotides or *CpG sites*), and is mediated by DNA methyl-transferases [6,7]. DNA methylation has been shown to play an important functional role in the cell, including involvement in DNA replication and gene transcription, with substantial downstream association with development, aging, and cancer [1-3,8-10].

CpG sites are under-represented in the human genome relative to their expected frequency as a result of being *mutation hotspots*, where the deamination of methylated cytosines encourages CpG sites to mutate to TpG sites [5,11]. Although CpG sites are mainly methylated across the mammalian genome [12], there are distinct, mostly unmethylated CG-rich regions called *CpG islands* (CGIs), which have a G+C content greater than 50*%* [5,11,13]. CGIs account for 1 to 2% of the genome and are often located in promoters and exonic regions in mammalian genomes [14,15]. Methylation patterns in CGIs that are in promoter regions, where most previous studies have focused attention, have recently been shown to differ from methylation patterns elsewhere, indicating a specific biological role for these promoter CGIs [12]. CGIs have been shown to co-localize with DNA regulatory elements such as transcription factor binding sites (TFBSs) [16-23] and DNA binding insulator proteins, such as CTCF, which insulate downstream DNA from upstream methylation activity [24]. Across the genome, DNA methylation levels have been shown to be dependent on context: methylation levels are relatively predictable within particular genomic regions. In particular, predictable levels of methylation have been observed in active chromatin marks [25-27] and cis-acting DNA regulatory elements [14,28]. Context-dependent methylation suggests cellular processes that regulate methylation and also provides clues as to how methylation may impact cellular phenotypes.

The non-uniform distribution of CpG sites across the human genome and the important role of methylation in cellular processes imply that characterizing genome-wide DNA methylation patterns is necessary for a better understanding of the regulatory mechanisms of this epigenetic phenomenon [29]. Recent advances in methylation-specific microarray and sequencing technologies have enabled the assay of DNA methylation patterns genome-wide at single base-pair resolution [29]. The current gold standard for quantifying single-site DNA methylation levels across a genome is whole-genome bisulfite sequencing (WGBS), which quantifies DNA methylation levels at ∼26 million (out of 28 million in total) CpG sites in the human genome [30-32]. However, WGBS is prohibitively expensive for most current studies, is subject to conversion bias, and is difficult to perform in particular genomic regions [29]. Other sequencing methods include methylated DNA immunoprecipitation sequencing, which is experimentally difficult and expensive, and reduced representation bisulfite sequencing, which assays CpG sites in small regions of the genome [29]. As an alternative, methylation microarrays, and the Illumina HumanMethylation450 BeadChip in particular, measure bisulphite-treated DNA methylation levels at ∼482,000 preselected CpG sites genome-wide [33]; however, these arrays assay less than 2*%* of CpG sites, and this percentage is biased to gene regions and CGIs. Quantitative methods are needed to predict methylation status at unassayed sites and genomic regions.

In this study, we examined measurements of methylation levels in 100 individuals using the Illumina 450K BeadChip [34]. Within these methylation profiles, we examined the patterns and correlation structure of the CpG sites, with attention to characterizing methylation patterns in CGI regions. Using features that include neighboring CpG site methylation status, genomic location, local genomic features, and co-localized regulatory elements, we developed a random forest (RF) classifier to predict single-CpG-site methylation levels genome-wide. Using this method, we were able to identify DNA regulatory elements that were especially predictive of DNA methylation levels at single CpG sites, providing hypotheses for experimental studies on mechanisms by which DNA methylation is regulated or leads to biological changes or disease phenotypes.

### Related work in DNA methylation prediction

Methylation status is a difficult epigenomic feature to characterize and predict because assayed DNA methylation marks are: (a) an average across the sampled cells, (b) specific to a cell type, (c) environmentally unstable and (d) not well correlated within a genomic locus [2,35,36]. Specific CpG sites may show differential methylation status across platforms, cell types, individuals or genomic regions [37,38]. A number of methods to predict methylation status have been developed (Additional file 1: Table S1). Most of these methods assume that methylation status is encoded as a binary variable, e.g., a CpG site is either methylated or unmethylated in an individual [28,39-45].

Related methods have often limited predictions to specific regions of the genome, such as CGIs [40-43,45,46]. These methods make predictions of average methylation status for windows of the genome instead of individual CpG sites (with one exception [38]). All of the studies that achieved prediction accuracy ≥90% [40,43,45,46] predicted average methylation status within CGIs or DNA fragments within CGIs. Most of the CpG sites in CGIs are unmethylated across the genome [12] – for example, 16*%* of CpG sites in CGIs in samples from the human brain were found to be methylated using a WGBS approach [47] – so it is not surprising that classifiers limited to these regions perform well. Studies extending prediction beyond CGIs uniformly achieved lower accuracies, ranging from 75*%* to 86*%*. Only two studies predicted methylation levels as a continuous variable: one study was limited to ∼400 bp DNA fragments instead of a genome-wide analysis [48], and the other used as prediction features the same CpG site in reference samples [38].

Across these methods, features that are used for DNA methylation prediction include: DNA composition (proximal DNA sequence patterns), predicted DNA structure (e.g., co-localized introns), repeat elements, TFBSs, evolutionary conservation (e.g., *PhastCons* [49]), single nucleotide polymorphisms (SNPs), GC content, Alu elements, histone modification marks, and functional annotations of nearby genes. Several studies used only DNA composition features [28,39,42,44,48]. Bock *et al.* used ∼700 features including DNA composition, DNA structure, repeat elements, TFBSs, evolutionary conservation, and number of SNPs [40]; Zheng *et al.* included ∼300 features including DNA composition, DNA structure, TFBSs, histone modification marks, and functional annotations of nearby genes [45]. One study used as features methylation levels from the same CpG sites in reference samples from different cell types [38]. The relative contribution of each feature to prediction quality is not quantified well within or across these studies because of the different methods and prediction objectives.

The majority of these methods are based on support vector machine (SVM) classifiers [28,38-41,43,45,46,48]. General non-additive interactions between features are not encoded when using linear kernels, which are used by most of these SVM-based classifiers. If a more sophisticated kernel is used, such as a radial basis function kernel, within the SVM-based approach, the contribution of each feature to prediction quality is not readily available. Three studies included alternative classification frameworks: one found that a decision tree classifier achieved better performance than an SVM-based classifier [46]. Another study found that a naive Bayes classifier achieved the best prediction performance [42]. A third study used a word composition-based encoding method [44].

Our method for predicting DNA methylation levels at CpG sites genome-wide differs from these current state-of-the-art classifiers in that it: (a) uses a genome-wide approach, (b) makes predictions at single-CpG-site resolution, (c) is based on a RF classifier, (d) predicts methylation levels *β* instead of methylation status *τ*, (e) incorporates a diverse set of predictive features, including regulatory marks from the ENCODE project, and (f) allows the quantification of the contribution of each feature to prediction. We find that these differences substantially improve the performance of the classifier and also provide testable biological insights into how methylation regulates, or is regulated by, specific genomic and epigenomic processes.

## Results

### Characterizing methylation patterns

DNA methylation profiles were measured in whole blood samples from 100 unrelated human participants by Illumina HumanMethylation450 BeadChips at single-CpG-site resolution for 482,421 CpG sites [50]. single-CpG-site methylation levels are quantified by *β*, the proportion of probes for this CpG site that are methylated, which is computed as the methylated probe intensity divided by the sum of both the methylated and unmethylated probe intensities; thus, *β* ranges from zero (the CpG site is unmethylated) to one (the CpG site is fully methylated). Within the single-CpG-site *β* values across individuals, we controlled for probe chip position, sample age, and sample sex. After these data were filtered and preprocessed (see Materials and methods), 394,354 CpG sites remained across the 22 autosomal chromosomes.

First, we examined the distribution of DNA methylation levels, *β*, at CpG sites on autosomal chromosomes across all 100 individuals. The majority of CpG sites were either *hypermethylated* or *hypomethylated* (levels of methylation that are consistently higher or lower than 0.5, respectively), with 48.2*%* of sites with *β*>0.7 and 40.4*%* of sites with *β*<0.3 (Additional file 1: Figure S1A). Using a cutoff of 0.5, across the methylation profiles and individuals, 54.8*%* of these CpG sites have a *methylated status* (*β*≥0.5). Across the individuals, we observed distinct patterns of DNA methylation levels in different genomic regions (Additional file 1: Figure S1B). Using CGIs labeled in the UCSC genome browser [51], we defined *CGI shores* as regions 0 to 2 kb away from CGIs in both directions and *CGI shelves* as regions 2 to 4 kb away from CGIs in both directions [34]. We found that CpG sites in CGIs were hypomethylated (81.2*%* of sites with *β*<0.3) and sites in non-CGIs were hypermethylated (73.2*%* of sites with *β*>0.7), while CpG sites in CGI shore regions had variable methylation levels following a U-shape distribution (39.0*%* of sites with *β*>0.7 and 46.2*%* of sites with *β*<0.3), and CpG sites in CGI shelf regions were hypermethylated (78.2*%* of sites with *β*>0.7). These distinct patterns reflect highly context-specific DNA methylation levels genome-wide.

**Figure 1:**
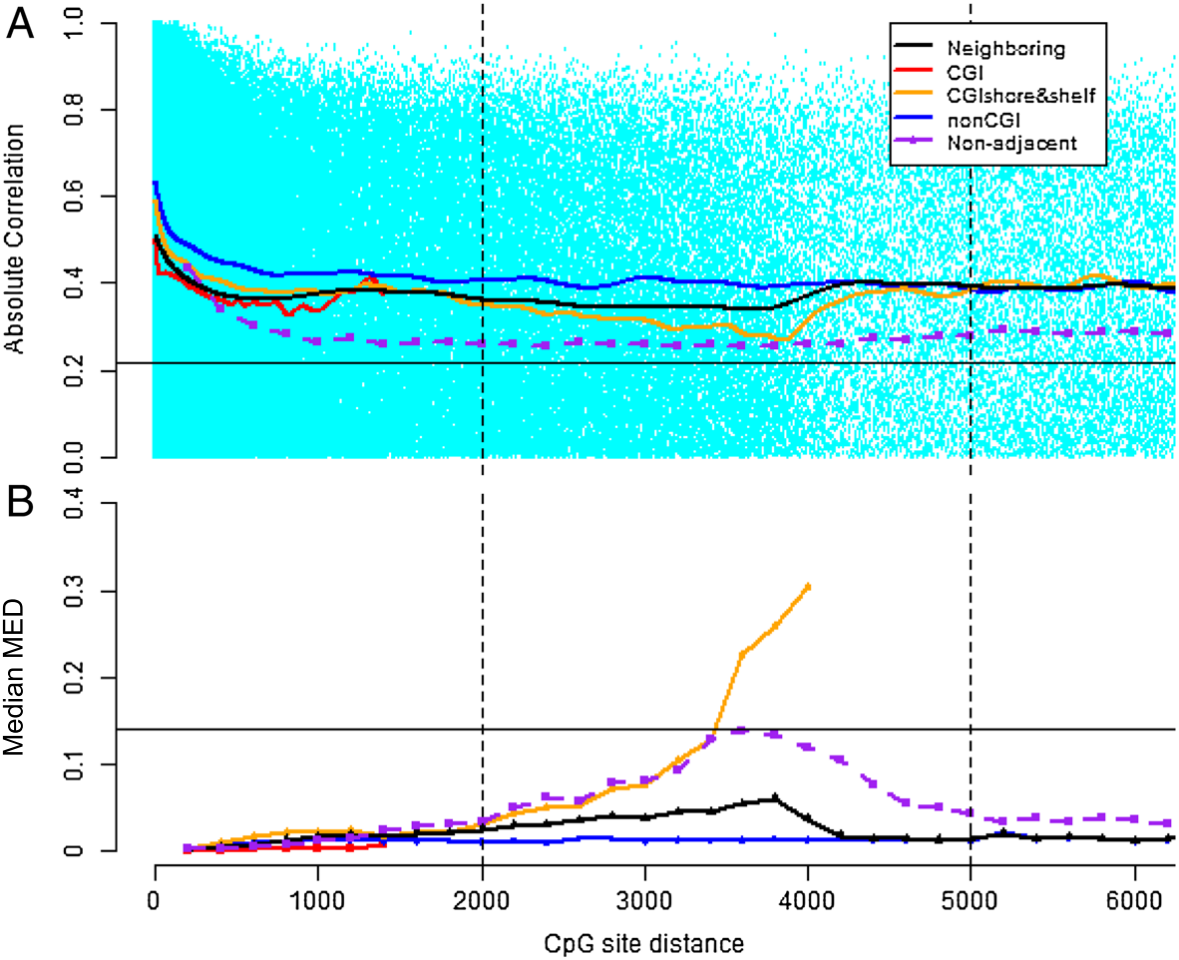
**Correlation of methylation levels between neighboring CpG sites.** The *x*-axis represents the genomic distance in bases between the neighboring CpG sites, or assayed CpG sites that are adjacent in the genome. Different colors and points represent subsets of the CpG sites genome-wide, including pairs of CpG sites that are not adjacent in the genome but that are the specified distance apart (*non-adjacent*). The CGI shore and shelf CpG sites are truncated at 4,000 bp, which is the length of the CGI shore and shelf regions. The solid horizontal line represents the background (absolute value correlation or mean squared Euclidean distance, MED) level from 50,000 pairs of CpG sites from different chromosomes. **(A)** Absolute value of the correlation between neighboring sites across all individuals (*y*-axis). The lines represent cubic smoothing splines fitted to the correlation data. **(B)** Median MED was calculated (*y*-axis) across pairs of CpG sites within the genomic distance window (*x*-axis). bp, base pair; CGI, CpG island; MED, mean squared Euclidean distance.

DNA methylation levels at nearby CpG sites have previously been found to be correlated (indicating possible co-methylation), particularly when CpG sites are within 1 to 2 kb from each other [35,36]. These methylation patterns stand in contrast with correlation among nearby genetic polymorphisms due to linkage disequilibrium, which often extends to large genomic regions from a few kilobases to >1 Mb [52]. We quantified the correlation of methylation levels *β* between neighboring pairs of CpG sites using the absolute value Pearson’s correlation across individuals. We found that correlation of methylation levels between *neighboring* (i.e., adjacent CpG sites in the genome that are both assayed) CpG sites decreased rapidly to approximately 0.4 within ∼400 bp, in contrast to sharp decays noted within 1 to 2 kb in previous studies with sparser CpG site coverage (Figure 1A) [35,36].

**Figure 2:**
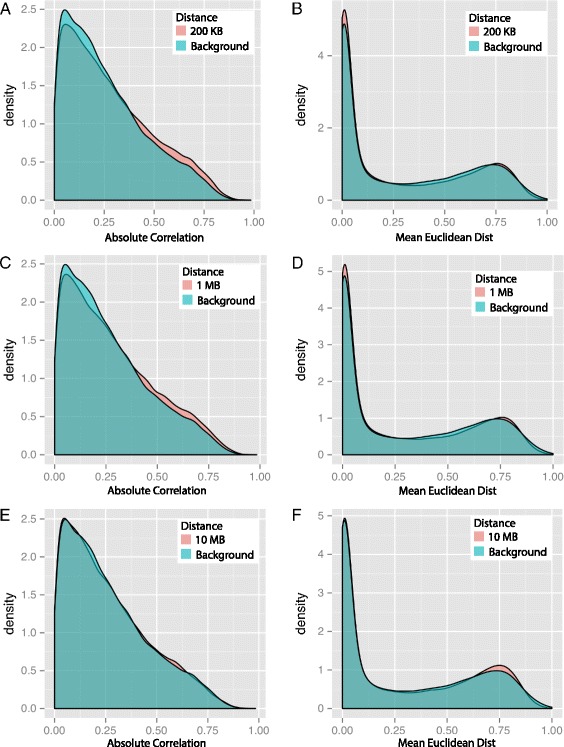
**Histogram of correlation and MED of methylation values between pairs of CpG sites.** The *x*-axes represent the correlation or MED of methylation values between pairs of CpG sites; the left column plots show the histogram of correlation of CpG sites within 200 kb **(A)**, 1 Mb **(C)** and 10 Mb **(E)**; the right column plots show the histogram of MED of CpG sites within 200 kb **(B)**, 1 Mb **(D)** and 10 Mb **(F)**. The distribution of the background is calculated by 50,000 random selected pairs of CpG sites and is shown in blue; The distributions of correlation and MED with corresponding distances are shown in pink. dist, distance; kb, kilobase, MB, megabase; MED, mean squared Euclidean distance.

To make this decay more precise, we contrasted the observed decay to the level of *background correlation* (0.22), which is the median absolute value Pearson’s correlation between the methylation levels of pairs of randomly selected pairs of CpG sites across chromosomes (Figure 1A). We found substantial differences in correlation between neighboring CpG sites versus randomly sampled pairs of CpG sites at matching distances, presumably because of the dense CpG tiling on the 450K array within CGI regions. Interestingly, the slope of the correlation decay plateaus after the CpG sites are approximately 400 bp apart (both for neighbors and for randomly sampled pairs at a matching distance). However, the distribution of correlation between pairs of CpG sites matches the distribution of background correlation even within 200 kb (Figure 2A, Additional file 1: Figure S2A). We found the rate of decay in the correlation to be highly dependent on genomic context; for example, for neighboring CpG sites in the same CGI shore and shelf region, correlation decreases continuously until it is well below the background correlation (Figure 1A). Because of the over-representation of CpG sites near CGIs on the 450K array, we see an increase in correlation as the distance between neighboring sites extends past the CGI shelf regions, where there is lower correlation with CGI methylation levels than we observe in the background. While this suggests that there may be types of methylation regulation that extend to large genomic regions, the pattern of extreme decay within approximately 400 bp across the genome indicates that, in general, methylation may be biologically manipulated within very small genomic windows. Thus, neighboring CpG sites may only be useful for prediction when the sites are sampled at sufficiently high densities across the genome.

We repeated these experiments using the mean squared Euclidean distance (MED) between pairs of CpG site levels to quantify patterns of decay of methylation within each individual, instead of across individuals, as is measured with the correlation analyses (see Materials and methods; Figure 1B, Figure 2B,D,F). In general, the MED trends echo the local patterns seen in the correlation analysis and also appear to be specific to a region. In CGI regions, the MED of neighboring sites was low and increased slowly with genomic distance. In contrast, MED in CGI shore and shelf regions increased rapidly to an MED higher than background MED (i.e., median MED between pairs of CpG sites within the same individual across chromosomes; 0.13), indicating that the edges of a single shore and shelf region are less predictive of each other than any two CpG sites at random. The individual-specific MED between neighboring sites (Additional file 1: Figure S2B) shows substantial deviation from the background distribution of MED at 200 kb relative to the correlation at this distance, indicating that there may be biological manipulation of methylation in larger genomic regions, but this manipulation may be specific to an individual, such as being driven by genetic variants or environmental effects. That said, for both MED and correlation distributions, we applied the Kolmogorov–Smirnov test to the background distribution and MED or correlation distributions at three genomic distances. We found that, in every case, the *P* values indicated that the null hypothesis – that the two samples came from the same underlying distribution – should be rejected. We found it difficult to reconcile the results of these tests against the relative histograms and quantile–quantile plots of the same samples (Figure 2, Additional file 1: Figure S2), which showed low levels of enrichment at high correlation and low MED in the non-background distributions.

**Figure 3:**
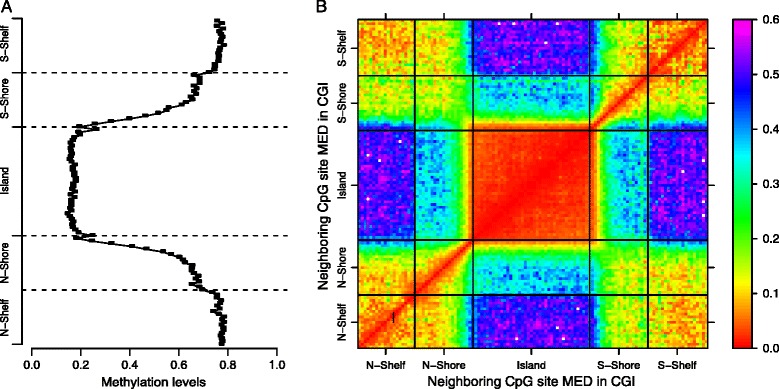
**Methylation levels, correlation within CGI.** Since each CGI is a different length, each CGI was split into 40 equal-sized windows, and methylation levels and correlation were averaged within each window. **(A)** The *x*-axis is the mean *β* value within a window in the CGI, CGI shore, or CGI shelf regions across all sites in all individuals with a window size of 100 bp. **(B)** Methylation values of each CpG site in a CGI, CGI shore or CGI shelf Oxford, were compared with all other sites in the same CGI using MED. The *x*-axis and *y*-axis represent the genomic position of each CGI with a scale of 1:100, i.e. one unit in the matrix represents 100 bp distance. The MED of each unit cell was calculated for all pair-wise CpG sites corresponding to that matrix position and averaged over the 100 individuals. bp, base pair; CGI, CpG island; MED, mean squared Euclidean distance.

As we observed that methylation patterns at neighboring CpG sites depend on genomic content, we further investigated methylation patterns within CGIs, CGI shores, and CGI shelves. Methylation levels at CGIs and CGI shelves were fairly constant genome-wide and across individuals – CGIs are hypomethylated and CGI shelves are hypermethylated – but CGI shores exhibit a reproducible but drastic pattern of change (Figure 3A). CpG sites in CGI shores have a monotone increasing pattern of methylation status from CGIs towards CGI shelves, and this pattern is symmetric in the CGI shores upstream and downstream of CGIs. We examined the MED between methylation status for pairs of CpG sites in these regions, and we found that MED within the CGI and within the CGI shelves is low, consistent with the variance we observed within DNA methylation profiles in these regions (Figure 3B). Additionally, we found that the MED between CpG sites in the shelves appears to increase as the sites are further away from the CGI on the shelf, suggesting a circular dependency in CpG site methylation across the ends of the shelf sequences. It is interesting that the CpG sites in the shore regions are substantially more predictive of CpG sites in the shelf regions than those in the CGI regions, although this may indicate a less precise delineation of the shore and shelf regions relative to the CGI and CGI shore delineation.

**Figure 4:**
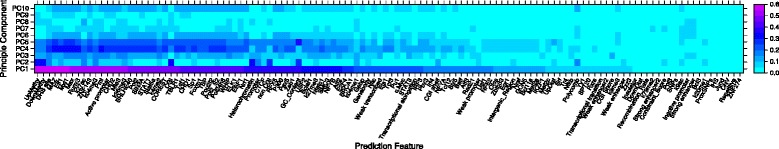
**Correlation matrix of prediction features with first ten PCs of methylation levels.** The *x*-axis corresponds to one of the 122 features; the *y*-axis represents PCs 1 through 10. Colors correspond to Pearson’s correlation, as shown in the legend. PC, principal component.

To quantify the amount of variation in DNA methylation explained by genomic context, we considered the correlation between genomic context and principal components (PCs) of methylation levels across all 100 samples (Figure 4). We found that many of the features derived from a CpG site’s genomic context appear to be correlated with the first principal component (PC1). The methylation status of upstream and downstream neighboring CpG sites and a co-localized DNAse I hypersensitive (DHS) site are the most highly correlated features, with Pearson’s correlation *r*=[0.58,0.59] (*P*<2.2×10^−16^). Ten genomic features have correlation *r*>0.5 (*P*<2.2×10^−16^) with PC1, including co-localized active TFBSs *ELF1* (ETS-related transcription factor 1), *MAZ* (Myc-associated zinc finger protein), *MXI1* (MAX-interacting protein 1) and *RUNX3* (Runt-related transcription factor 3), and co-localized histone modification trimethylation of histone H3 at lysine 4 (H3K4me3), suggesting that they may be useful in predicting DNA methylation status (Additional file 1: Figure S3). That said, the features themselves are well correlated; for example, active TFBS *ELF1* is highly enriched within DHS sites (*r*=0.67,*P*<2.2×10^−16^) [53,54].

### Binary methylation status prediction

These observations about patterns of DNA methylation suggest that correlation in DNA methylation is local and dependent on genomic context. Thus, prediction of DNA methylation status based only on methylation levels at neighboring CpG sites may not perform well, especially in sparsely assayed regions of the genome. Using prediction features, including neighboring CpG site methylation levels and features characterizing genomic context, we built a classifier to predict binary DNA methylation status. *Status*, which we denote using *τ*_*i*,*j*_∈{0,1} for *i*∈{1,…,*n*} samples and *j*∈{1,…,*p*} CpG sites, indicates no methylation (0) or complete methylation (1) at CpG site *j* in sample *i*. We computed the status of each site from the *β*_*i*,*j*_ variables: $\tau _{i,j} = \mathbb {1}[\beta _{i,j} > 0.5]$. For each sample, there were 378,677 CpG sites with neighboring CpG sites on the same chromosome, which we used in these analyses.

The 124 features that we used for DNA methylation status prediction fall into four different classes (see Additional file 1: Table S2 for a complete list). For each CpG site, we include the following feature sets:

- *neighbors*: genomic distances, binary methylation status *τ* and levels *β* of one upstream and one downstream neighboring CpG site (CpG sites assayed on the array and adjacent in the genome)
- *genomic position*: binary values indicating co-localization of the CpG site with DNA sequence annotations, including promoters, gene body, intergenic region, CGIs, CGI shores and shelves, and nearby SNPs
- *DNA sequence properties*: continuous values representing the local recombination rate from HapMap [55], GC content from ENCODE [56], integrated haplotype scores (iHSs) [57], and genomic evolutionary rate profiling (GERP) calls [58]
- *cis-regulatory elements*: binary values indicating CpG site co-localization with cis-regulatory elements (CREs), including DHS sites, 79 specific TFBSs, ten histone modification marks and 15 chromatin states, all assayed in the GM12878 cell line, the closest match to whole blood [56]

We used a RF classifier, which is an ensemble classifier that builds a collection of bagged decision trees and combines the predictions across all of the trees to produce a single prediction. The output from the RF classifier is the proportion of trees in the fitted forest that classify the test sample as a 1, $\hat {\beta }_{i,j}\in [0,1]$ for *i*={1,…,*n*} samples and *j*={1,…,*p*} CpG sites assayed. We thresholded this output to predict the binary methylation status of each CpG site, $\hat {\tau }_{i,j} \in \{0,1\}$, using a cutoff of 0.5. We quantified the generalization error for each feature set using a modified version of repeated random subsampling (see Materials and methods). In particular, we randomly selected 10,000 CpG sites genome-wide for the training set, and we tested the fitted classifier on all held-out sites in the same sample. We repeated this ten times. We quantified prediction accuracy, specificity, sensitivity (recall), precision (1− false discovery rate), area under the receiver operating characteristic (ROC) curve (AUC), and area under the precision–recall curve (AUPR) to evaluate our predictions (see Materials and methods).

**Figure 5:**
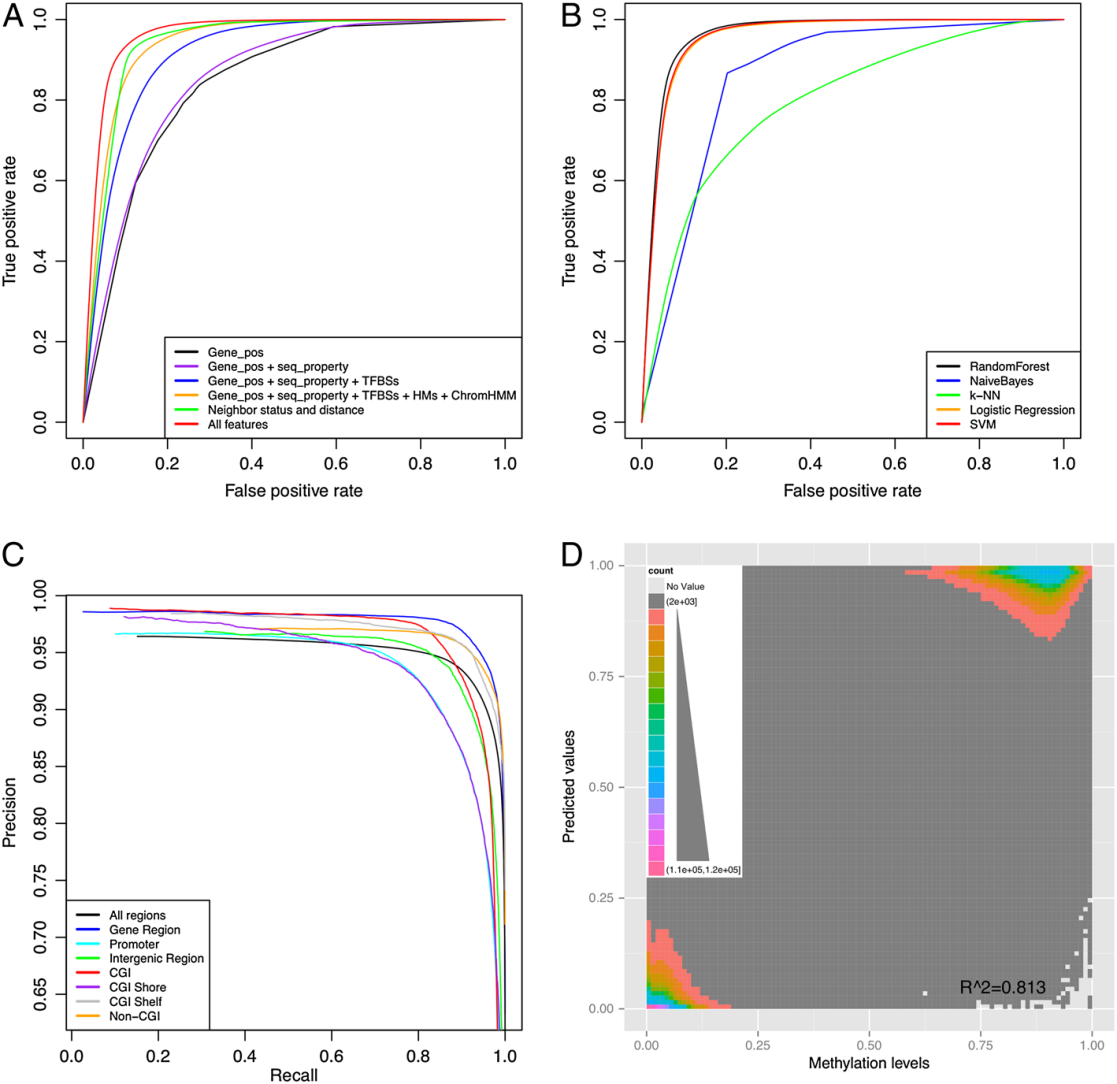
**Prediction performance of methylation status and level.** **(A)** ROC curves of cross-genome validation of methylation status prediction. Colors represent classifier trained using feature combinations specified in the legend. Each ROC curve represents the average false positive rate and true positive rate for prediction on the held-out sets for each of the ten repeated random subsamples. **(B)** ROC curves for different classifiers. Colors represent prediction for a classifier denoted in the legend. Each ROC curve represents the average false positive rate and true positive rate for prediction on the held-out sets for each of the ten repeated random subsamples. **(C)** Precision–recall curves for region-specific methylation status prediction. Colors represent prediction on CpG sites within specific genomic regions as denoted in the legend. Each precision–recall curve represents the average precision–recall for prediction on the held-out sets for each of the ten repeated random subsamples. **(D)** Two-dimensional histogram of predicted methylation levels versus experimental methylation levels. *x*- and *y*-axes represent assayed versus predicted *β* values, respectively. Colors represent the density of each matrix unit, averaged over all predictions for 100 individuals. CGI, CpG island; Gene_pos, genomic position; k-NN, *k*-nearest neighbors classifier; ROC, receiver operating characteristic; seq_property, sequence properties; SVM, support vector machine; TFBS, transcription factor binding site; HM, histone modification marks; ChromHMM, chromatin states, as defined by ChromHMM software [107].

Using 122 features (excluding *β* for one upstream and one downstream neighboring CpG site but including status *τ*) and considering all CpG sites with two neighboring CpG sites in our data, we achieved an accuracy of 91.9*%* and an AUC of 0.96 (Figure 5A). We considered the role of each subset of features (Table 1). For example, if we only included *genomic position* features, the classifier had an accuracy of 78.6*%* and AUC of 0.85. Including *DNA sequence properties* and TFBS features increased the accuracy to 85.7*%* and the AUC to 0.92. When we included all classes of features except for neighbors, the classifier achieved an accuracy of 89.0*%* and an AUC of 0.94, a significant improvement in prediction from only considering *genomic position* features (*t*-test; *P*=7.75×10^−23^). These results suggest that TFBSs, histone modifications, and chromatin state are predictive of DNA methylation. However, we also found that the genomic context features improved prediction significantly over using only the neighbor features, which has an accuracy of 90.7*%* and an AUC of 0.94 (*t*-test; *P*=3.45×10^−18^).

**Table 1:** **Performance of methylation status prediction using different prediction models**

**Feature set**	**Features**	**Accuracy (%)**	**AUC**	**Specificity (%)**	**Sensitivity (%)**	**MCC**	***R***	**RMSE**
Gene_pos ^a^	9	78.6	0.85	72.5	83.6	0.57	0.61	0.39
Gene_pos + seq_property ^b^	13	79.5	0.86	71.6	85.9	0.58	0.66	0.34
Gene_pos + seq_property + TFBSs	93	85.7	0.92	78.4	91.7	0.71	0.80	0.29
Gene_pos + seq_property + CREs ^c^	118	89	0.94	83.9	93.3	0.78	0.86	0.23
Neighbor CpG methylation status + distance ^d^	4	90.7	0.94	87.2	93.5	0.81	0.87	0.24
All features	122	91.9	0.96	87.9	95.1	0.84	0.90	0.19

^a^Genomic position features including gene region status (promoter, gene body, and intergenic region), CGI status (CGI, CGI shore, CGI shelf, and non-CGI), and proximal SNPs. ^b^DNA sequence properties include GC content, recombination rate, conservation score, and iHSs. ^c^CREs include TFBSs, DHS sites, histone modifications and chromatin state segmentation. ^d^Genomic distance between neighboring CpG sites. AUC, area under ROC curve; CGI, CpG island; CRE, cis-regulatory element; iHS, integrated haplotype score; MCC, Matthew’s correlation coefficient; RMSE, root-mean-square error; SNP, single nucleotide polymorphism; TFBS, transcription factor binding site.

#### Cross-sample prediction

To determine how predictive methylation profiles were across samples, we quantified the generalization error of our classifier genome-wide across individuals. In particular, we trained our classifier on 10,000 sites from one individual, and predicted methylation status for all CpG sites for the other 99 individuals. The classifier’s performance was highly consistent across individuals (Additional file 1: Figure S4), suggesting that individual-specific covariates – different proportions of cell types, for example – do not limit prediction accuracy. Aware of the unbalanced proportion of female and male samples in our study, we further investigated prediction performance across sex. The classifier’s performance is highly consistent when training on females and predicting CpG site methylation status in males, and vice versa (Additional file 1: Figure S5).

To test the sensitivity of our classifier to the number of CpG sites in the training set, we investigated the prediction performance for different training set sizes. We found that training sets with greater than 1,000 CpG sites had fairly similar performance (Additional file 1: Figure S6). Throughout these experiments, we used a training set size of 10,000, to strike a balance between sufficient numbers of training samples and computational tractability.

#### Cross-platform prediction

To quantify classification across platform and cell-type heterogeneity, we investigated the classifier’s performance on WGBS data [59,60]. In particular, we categorized each CpG site in a WGBS sample based on whether that CpG site was assayed on the 450K array (*450K site*) or not (*non 450K site*); *neighboring sites* in the WGBS data are sites that are adjacent on the genome when both are 450K sites. We use one WGBS sample from b-cells, which will match some proportion of each whole blood sample; we note that the 450K array whole blood samples will contain heterogeneous cell types in contrast to the WGBS data. Overall, we see a much higher proportion of hypomethylated CpG sites on the 450K array relative to the WGBS data (Additional file 1: Figure S7) because of the disproportionate representation of hypomethylated CpG sites within CGIs on the 450K array.

First, we investigated cross-platform prediction, training our classifier on a 450K array sample and testing on WGBS data. We trained the classifier on 10,000 CpG sites in the 450K array samples, and then we tested on 100,000 CpG sites in WGBS data twice – once restricting the test set to 450K sites and once restricting the test set to non 450K sites. We repeated this experiment ten times. Next, we performed the same experiment but trained and tested on the WGBS data. Because the proportion of hypomethylated and hypermethylated sites was imbalanced for CpG sites not on the 450K array, we used a precision–recall curve instead of a ROC curve to measure the prediction performance [61]. We used all 122 features and considered prediction of inverse CpG status ${\hat {\tau }} = -(\tau - 1)$ in this experiment, to assess the quality of the predictions for the less frequent class of hypomethylated CpG sites.

**Figure 6:**
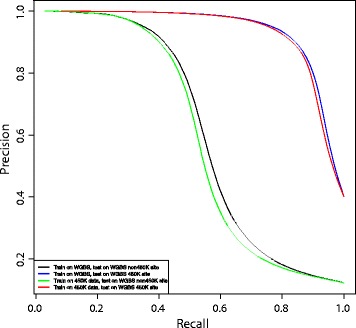
**Prediction performance on WGBS data and cross-platform prediction.** Precision–recall curves for cross-platform and WGBS prediction. Each precision–recall curve represents the average precision–recall for prediction on the held-out sets for each of the ten repeated random subsamples. WGBS, whole-genome bisulfite sequencing.

Trained on 450K array data and tested on WGBS 450K sites, our RF classifier achieved an accuracy of 89.3*%*; trained on 450K array data and tested on WGBS non 450K sites, our RF classifier achieved an accuracy of 92.2*%* (Figure 6; Table 2). Training and testing exclusively on WGBS data showed a similar performance, with an accuracy of 90.0*%* for CpG sites in the 450K sites and 92.4*%* for CpG sites in the non 450K sites (Figure 6). Predictions for CpG sites in non 450K sites had lower precision at high recall rates because it is more difficult to predict unmethylated sites in the sequencing data as there are many more unmethylated CpG sites. These results suggest that our RF classifier is able to generalize across platforms and methylation assay types.

**Table 2:** **Performance of methylation prediction using whole-genome bisulfite sequencing data**

**Training set**	**Test set**	**Accuracy (%)**	**Precision (%)**	**Recall (%)**	**TN**	**FN**	**TP**	**FP**	***R***	**RMSE**
WGBS 450K sites	WGBS non 450K sites	92.4	86.5	44.5	78264	6095	4890	764	0.64	0.24
WGBS 450K sites	WGBS 450K sites	90	91.8	82.3	51260	6374	29726	2653	0.86	0.23
450K data	WGBS non 450K sites	92.2	88.5	41.4	78437	6442	4543	591	0.62	0.23
450K data	WGBS 450K sites	89.3	93.0	79.3	51762	7465	28635	2151	0.84	0.24

FN, false negatives; FP, false positives; RMSE, root-mean-square error; TN, true negatives; TP, true positives; WGBS, whole-genome bisulfite sequencing.

### Comparison of random forest classifier with other classifiers

We compared the prediction performance of our RF classifier with several other classifiers that have been widely used in related work (Table 3). In particular, we compared our prediction results from the RF classifier with those from a SVM classifier with a radial basis function kernel, a *k*-nearest neighbors classifier (k-NN), logistic regression, and a naive Bayes classifier. We used identical feature sets for all classifiers, including all 122 features used for prediction of methylation status with the RF classifier. We quantified performance using repeated random resampling with identical training and test sets across classifiers.

**Table 3:** **Performance of methylation status prediction using different classifiers**

**Classifier**	**Accuracy (%)**	**AUC**	**Specificity (%)**	**Sensitivity (%)**	**MCC**
k-NN	73.2	0.80	72.6	73.7	0.46
Naive Bayes	80.8	0.91	64.4	94.2	0.62
Logistic regression	91.1	0.96	87.3	94.1	0.82
SVM	91.3	0.96	86.6	95.1	0.82
Random forest	91.8	0.96	87.9	95.1	0.84

AUC, area under the receiver operating characteristic curve; k-NN, *k*-nearest neighbors classifier; MCC, Matthew’s correlation coefficient; SVM, support vector machine.

We found that the k-NN classifier showed the worst performance on this task, with an accuracy of 73.2*%* and an AUC of 0.80 (Figure 5B). The naive Bayes classifier showed better accuracy (80.8*%*) and AUC (0.91). Logistic regression and the SVM classifier both showed good performance, with accuracies of 91.1*%* and 91.3*%* and AUCs of 0.96*%* and 0.96*%*, respectively. We found that our RF classifier showed significantly better prediction accuracy than logistic regression (*t*-test; *P*=3.8×10^−16^) and the SVM (*t*-test; *P*=1.3×10^−13^). We note also that the computational time required to train and test the RF classifier was substantially less than the time required for the SVM, k-NN (test only), and naive Bayes classifiers. We chose RF classifiers for this task because, in addition to the gains in accuracy over SVMs, we were able to quantify the contribution to prediction of each feature, which we describe below.

### Region-specific methylation prediction

Studies of DNA methylation have focused on methylation within promoter regions, restricting predictions to CGIs [40,41,43-46,48]; we and others have shown DNA methylation has different patterns in these genomic regions relative to the rest of the genome [12], so the accuracy of these prediction methods outside of these regions is unclear. Here we investigated regional DNA methylation prediction for our genome-wide CpG site prediction method restricted to CpGs within specific genomic regions (Additional file 1: Table S3). For this experiment, prediction was restricted to CpG sites with neighboring sites within 1 kb distance because of the small size of CGIs.

Within CGI regions, we found that predictions of methylation status using our method had an accuracy of 98.3*%*. We found that methylation level prediction within CGIs had an *r*=0.94 and a root-mean-square error (RMSE) of 0.09. As in related work on prediction within CGI regions, we believe the improvement in accuracy is due to the limited variability in methylation patterns in these regions; indeed, 90.3*%* of CpG sites in CGI regions have *β*<0.5 (Additional file 1: Table S4). Conversely, prediction of CpG methylation status within CGI shores had an accuracy of 89.8*%*. This lower accuracy is consistent with observations of robust and drastic change in methylation status across these regions [62,63]. Prediction performance within various gene regions was fairly consistent, with 94.9*%* accuracy for predictions of CpG sites within promoter regions, 93.4*%* accuracy within gene body regions (exons and introns), and 93.1*%* accuracy within intergenic regions. Because of the imbalance of hypomethylated and hypermethylated sites in each region, we evaluated both the precision–recall curves and ROC curves for these predictions (Figure 5C and Additional file 1: Figure S8).

### Predicting genome-wide methylation levels across platforms

CpG methylation levels *β* in a DNA sample represent the average methylation status across the cells in that sample and will vary continuously between 0 and 1 (Additional file 1: Figure S9). Since the Illumina 450K array measures precise methylation levels at CpG site resolution, we used our RF classifier to predict methylation levels at single-CpG-site resolution. We compared the prediction probability (${\hat \beta }_{i,j} \in \left [0,1\right ]$) from our RF classifier (without thresholding) with methylation levels (*β*_*i*,*j*_∈[0,1]) from the array, and validated this approach using repeated random subsampling to quantify generalization accuracy (see Materials and methods). Including all 122 features used in methylation status prediction, but modifying the neighboring CpG site methylation status *τ* to be continuous methylation levels *β*, we trained our RF classifier on 450K array data and evaluated the Pearson’s correlation coefficient (*r*) and RMSE between experimental and predicted methylation levels (Table 1; Figure 5D). We found that the experimentally assayed and predicted methylation levels had *r*=0.90 and RMSE =0.19. The correlation coefficient and the RMSE indicate good recapitulation of experimentally assayed levels using predicted methylation levels across CpG sites.

We quantified the performance of methylation level prediction on WGBS data. We trained on CpG sites from the 450K array, and tested the classifier on CpG sites from the WGBS data, both restricted to CpG sites in the 450K sites set and restricted to CpG sites in the non 450K sites set. We achieved different correlations (*r*=0.62 and 0.84, *P*<2.2×10^−16^) but similar RMSE (0.23 and 0.24, *P*=3×10^−16^) when predicting methylation levels for CpG sites in the 450K sites set and CpG sites in the non 450K sites set, respectively, in WGBS data. We suspect that the performance difference between the two experiments reflected in the correlation coefficients may be due to the overabundance of CpG sites from CGIs included on the 450K array and, correspondingly, in the 450K sites set of CpG sites.

### Feature importance for methylation prediction

**Figure 7:**
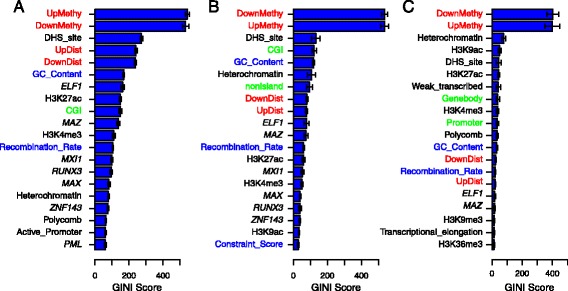
**Top 20 most important features by Gini index.** Gini index of the top 20 features for prediction in different genomic regions. Colors represent different types of features: neighbors in red, genomic position in green, sequence properties in blue and CREs in black. **(A)** Gini index for whole-genome prediction. **(B)** Gini index for prediction in promoter regions. **(C)** Gini index for prediction in CGIs. CGI, CpG island; CRE, cis-regulatory element; DHS, DNAse I hypersensitive; UpMethyl, upstream CpG site; DownMethyl, downstream CpG site; UpDist, distance in bases to the upstream CpG site; DownDist, distance in bases to the downstream CpG site.

We evaluated the contribution of each feature to overall prediction accuracy, as quantified by the Gini index. In the RF classifier, the *Gini index* measures the decrease in *node impurity*, or the relative entropy of the observed positive and negative examples before and after splitting the training samples on a single feature, of a given feature over all trees in the trained RF. We computed the Gini index for each of the 122 features from the trained RF classifier for predicting methylation status. Our analysis confirmed that the upstream and downstream neighboring CpG site methylation statuses are the most important features for prediction (Additional file 1: Table S5, Figure 7). When we restrict prediction to promoter or CGI regions, the Gini score of the neighboring site status features increased relative to other features, echoing our observation that the non-neighbor feature sets are less useful when a CpG site’s neighbors are nearby, and thus more informative. In contrast, we found that the Gini index of the genomic distance to the neighboring CpG site feature decreased, suggesting that neighboring genomic distance is an important feature to consider when some neighbors are more distant and correspondingly less predictive.

The CRE features also have variable Gini indices across experiments. We found that DHS sites are strongly predictive of an unmethylated CpG site; the DHS site feature has the third most significant Gini index across these experiments. This observation is consistent with a previous study showing that CpG sites in DHS sites tend to be unmethylated [64]. GC content, which was also ranked highly based on Gini index, may have a substantial contribution to prediction as a proxy for other important features, such as CGI status and CpG density. We found that the feature rankings based on Gini index differed when predicting methylation status in specific genomic regions (Figure 7), implying context-specific DNA methylation mechanisms.

When predicting methylation status in arbitrary regions, several transcription factors (TFs) and histone modifications were among the most highly ranked features across experiments. Some of these CREs have a reported association with DNA methylation, including *ELF1*, *RUNX3*, *MAZ*, *MXI1*, and *MAX*. Indeed, the ETS-related transcription factor (*ELF1*) has been shown to be over-represented in methylated regions, associating DNA methylation with hematopoiesis in hematopoietic stem cells [65]. *RUNX3* (Runt-related transcription factor 3), a strong tumor suppressor associated with diverse tumor types, has been suggested to be associated with cancer development through regulating global DNA methylation levels [66-71]. *RUNX3* expression is associated with aberrant DNA methylation in adenocarcinoma cells [70], primary bladder tumor cells [68], and breast cancer cells [69]. For another tumor suppressor transcription factor, *MXI1* (MAX-interacting protein 1), expression levels (specifically, lack of expression) have been reported to be associated with promoter methylation levels and neuroblastic tumorigenesis [72]. It has been suggested that suppression of *MAZ* (Myc-associated zinc finger protein) may be associated with DNA methyltransferase I, the key factor for *de novo* DNA methylation [73,74]. *MXI1* and *MAX* (Myc-associated factor X) both interact with c-Myc (myelocytomatosis oncogene), a well-characterized oncogene, which has been shown to be methylation sensitive, meaning that the TF motifs contain CpG sites and, thus, TF binding is sensitive to methylation status at those sites [75]. This suggests a potential regulatory relationship between *MAX*, *MXI1*, and DNA methylation that may extend to downstream cancer tumor development.

The association between specific histone modifications and DNA methylation is poorly understood. A previous study suggested that high H3K4 methylation and H3 acetylation are associated with *MYC* recognition [76], suggesting regulatory relationships among DNA methylation, histone modification, and transcription factor binding. Our results suggest that further work is needed to clarify this relationship, as a subset of histone modifications appear to be predictive of methylation status.

We found that the correlation between a binary feature and PC1 is proportional to the Gini index of that feature (Figure 4 and Additional file 1: Table S5). The variation in the Gini index rankings for CREs varied more than we expected based on the other features (Additional file 1: Figure S10). CREs that co-occur with CpG sites more often tend to be more important for prediction, according to the Gini index. We found that the Gini index of a binary feature has a log linear relationship with the number of co-occurrences of that binary feature with CpG sites in the data set: the more often a CpG site in the training data co-occurred with a CRE, the higher the Gini index rank of that CpG site (Additional file 1: Figure S10). There were several outliers to this trend, including co-localization with bound *POL3* (RNA polymerase III), C-fos (a proto-oncogene), and histone modifications H3K9ac and H4K20me. These features were less important than we would predict using the fitted linear regression model of log Gini index. This trend limits the strong conclusions that associate specific CREs with DNA methylation biochemically from a high Gini index rank for that CRE; it may be that there are general relationships between CREs and CpG sites that we are learning, but a relatively high CRE frequency in these data may artificially inflate the rank of that CRE in comparison to the others (Additional file 1: Figure S10). Most CpG sites within TFBSs have low average methylation levels (Additional file 1: Table S4). Several TFBSs have disproportionately high average methylation levels, for example, *ZNF274* (Zinc-finger protein 274) and *JunD* (Jun D proto-oncogene); however, both of these outliers also have a low co-occurrence frequency with CpG sites in these data, suggesting that this finding may be an artifact.

## Discussion

We characterized genome-wide and region-specific patterns of DNA methylation. We performed these characterizations based on summary statistics instead of a model-based analysis, which may lead to less dramatic region-specific methylation patterns than in our study (L Pachter, personal communication). These region-specific patterns raise additional questions, including how these observations may resolve or at least suggest causal relationships between methylation and other genomic and epigenomic processes. Since there are SNP associations with complex traits, it is likely that the genotype drives associated processes rather than the other way around; the causal relationship is established by inductive logic, since it is biologically difficult to perform site-specific mutation. The dynamic nature of CpG site methylation means that no such causal relationship can be established inductively; however, experiments can be designed to establish the impact of changing the methylation status of a CpG site [77,78]. Conditional analyses, such as those developed for DNA, may prove to be illuminating for epigenomics [79,80], but the current data are still difficult to interpret. For example, does a TFBS containing a CpG site prevent methylation when a transcription factor is actively bound, or does a methylated CpG site in a TFBS prevent a TF from binding to that site?

We built a RF predictor of DNA methylation levels at CpG site resolution. In our comparison between an RF classifier and alternative classifiers, we found that improvements of the RF classifier include better prediction, especially in sparsely sampled genomic regions, and biological interpretability, which comes from the ability to readily extract information about the importance of each feature in prediction. An additional benefit of using cell-type-specific features (i.e., CREs) is that the predictions are robust to differential methylation across cell types [81,82]. The accuracy results for predictions based on this model are promising, in particular the cross-cell-type heterogeneity and cross-platform performance, and suggest the possibility of imputing CpG site methylation levels genome-wide in the future using WGBS samples as reference. For example, if we assay a set of individuals in an epigenome-wide association study on the Illumina 450K array, we may be able to impute the missing genome-wide CpG sites up to WGBS assays. We are still far from the prediction accuracies currently expected for SNP imputation for downstream use in genome-wide association studies; however, in imputation we would include CpG site-specific methylation levels from reference samples, instead of predicting methylation levels in a site-independent way [38,83]. Our cross-sample analysis illustrates that including methylation profiles from other individuals as reference may improve accuracies substantially. However, because of biological, batch, and environmental effects on DNA methylation, it is possible that precise imputation will require a much larger reference panel relative to DNA imputation. As in genome-wide association studies, all of these imputation methods will fail to predict rare or unexpected variants [84], which may hold a substantial proportion of association signal for both genome-wide and epigenome-wide association studies [85-87]. This work raises the additional question, then, of how best to sample CpG sites across the genome given the methylation patterns and the possibility of imputation; for example, it may be sufficient to assay a single CpG site within a CGI and impute the others, given the high correlation between methylation values in CpG sites within the same CGI.

We identified genomic and epigenomic features that were most predictive of methylation status for co-located CpG sites. The biological functions of CGI shore and shelf regions, and in particular the impact of methylation in these regions, are mostly unknown; however, it has been shown there is substantial DNA methylation variation in CGI shore regions relative to other regions in the genome, and these alterations may contribute to cancer development and tissue differentiation [62,63]. We hope to characterize the role of CGI shore and shelf regions better with respect to enrichment of particular regulatory elements in the future to understand the cellular role of these regions and the specific, curious pattern of methylation found within them.

One particularly important driver of methylation that we do not study carefully here is methylation quantitative trait loci (QTLs), or genetic drivers of methylation [35,88,89]. There is substantial work on the enrichment of methylation QTLs within SNPs and genetic loci that appear to regulate gene transcription levels (expression quantitative trait loci, or *eQTLs*), DHS site status (dsQTLs), and others [35,64,88,90-92]. The characterizations described here lead us to consider identifying QTLs associated with deviations from CRE-specific methylation patterns instead of single CpG sites, as has been done with methylation in CGI shore regions and associations with cancer [63].

## Conclusions

We investigated genome-wide methylation in 100 individuals profiled using the Illumina 450K array. We identified patterns of correlation in DNA methylation at CpG sites specific to CGIs, CGI shores, and non-CGIs, quantifying the variability within CGI shore regions and a pattern of correlation across the shelf regions by which correlation increases with distance. We built a RF classifier to predict methylation as a binary status and as a continuous level at single-CpG-site accuracy, using as features neighboring CpG site information, genomic position, DNA sequence properties, and CRE co-location information. We found that our RF-based method outperformed alternative methylation status classifiers and produces interpretable results. We found that the accuracy of our classifier remained high when predicting methylation status in WGBS data, and across samples. Our approach quantifies features that are most predictive of CpG status: we found that neighboring CpG site methylation levels, location in a CGI, and co-localized DHS sites and specific transcription factor binding sites were most predictive of DNA methylation levels. We identified several TFBSs, including *ELF1*, *MAZ*, *MXI1*, and *RUNX3*, and histone modifications that are highly predictive of methylation levels in whole blood. These predictive features may play a mechanistic role in methylation, either in regulating the methylation of CpG sites or as a downstream partner in modifying the cellular phenotype.

## Materials and methods

### DNA methylation data

Illumina HumanMethylation450 array data were obtained for 100 unrelated human participants from the TwinsUK cohort [93]. All participants provided written informed consent in accordance with local ethics research committees. The 100 individuals were adult unselected volunteers and included 97 female and three male participants (age range 27 to 78). Whole blood was collected and DNA was extracted using standard protocols.

Illumina HumanMethylation450 array (Illumina 450K) measured the DNA methylation values for more than 482,000 CpG sites per individual at single-nucleotide resolution. Genomic coverage includes 99*%* of reference sequence genes, with an average of 17 CpG sites per gene region distributed across the promoter, 5^′^ UTR, first exon, gene body, and 3^′^ UTR, and 96*%* of CGIs [34,94].

Methylation values for each CpG site are quantified by the term *β*, which is the fraction of methylated bead signal over the sum of the methylated and unmethylated bead signals:

(1) $$ \beta =\frac{\max (\text{Methy},0)}{\max (\text{Methy},0)+\max (\text{Unmethy},0)+\alpha}  $$

where Methy represents the signal intensity of the methylated probe and Unmethy represents the signal intensity of the unmethylated probe. The quantity *β* ranges from 0 (unmethylated) to 1 (fully methylated).

Data quality control was implemented using R [95] (version 2.15.3). We removed 17,764 CpG sites for which probes mapped to multiple loci in the human genome reference sequence. CpG sites that are SNPs, that had missing values, or that had detection *P*>0.01 were excluded. Methylation data from probes mapping to the X and Y chromosomes were excluded. We were left with 394,354 CpG sites from 100 individuals in downstream analyses. We normalized the data as follows. Within the methylation level data, we controlled for array number, probe position on the array, age, and sex by taking the residual from a fitted linear regression model. The sum of residuals and intercepts of each site was scaled to [0,1] by truncating all sites with values larger than 1 to 1 and all sites with values smaller than 0 to 0. We assessed data quality to identify sample outliers and batch effects using principal component analysis (PCA) [96] across individuals and CpG sites; no obvious outliers were identified.

We downloaded the WGBS data (BED files) from NCBI Gene Expression Omnibus (GEO) [GEO:GSE46644], sample GSM791827 [59,60]. CD19+ B cells were purified from peripheral blood collected from one healthy female donor. Bisulfite sequencing and read mapping processes were described in previous work [60]. The methylation levels for each CpG site were quantified by the ratio of the number of methylated and the total reads at each CpG site. Only CpG sites with greater than 5 × coverage were included. Methylation level data from the X and Y chromosomes were excluded. After quality control, there were 10,000,890 CpG sites in the WGBS data. Because we used only a single sample, we did not control for PCs.

### Correlation and principal component analysis

The statistical analyses were implemented using R and Bioconductor [97] (version 2.15.3). Methylation correlations between CpG sites were assessed by the absolute value of Pearson’s correlation coefficient and MED:

(2) $$ \text{MED} = \frac{\sum_{i=1}^{n}(x_{i,j}-x_{i,k})^{2}}{n},  $$

where *x*_*i*,*j*_ and *x*_*i*,*k*_ represent the methylation values of the two CpG sites being compared *j* and *k*, and *n* represents the number of samples in the comparison. For neighboring CpG sites, pairs of CpG sites assayed on the array that were adjacent in the genome were sampled; the genomic distance between the pairs of CpG sites were within the range *x*−200 bp to *x* bp, where *x*∈{200,400,600,…,6,000}. The correlation and MED of a 200-bp window was not computed, as there were too few CpG sites. The non-adjacent pair correlation or MED values are the average absolute value correlation or MED of 5,000 pairs of CpG sites that were not immediate neighbors with their genomic distances in the same range as for the adjacent CpG sites.

We performed PCA on methylation values of CpG sites by computing the eigenvalues of the covariance matrix of a subsample of CpG sites using the R function svd. Among the 378,677 CpG sites that have complete feature information, 37,868 sites (every tenth CpG site) were sampled along the genome across all autosomal chromosomes. Absolute value Pearson’s correlation was calculated between each feature and the first ten PCs. PCA was performed by plotting the PC biplot (scatterplot of first two PCs), colored by feature status of each CpG site, and by computing the Pearson correlation between the PCs and the feature status across CpG sites.

### Random forest and comparison classifier

We used the randomForest package in R in the implementation of the RF classifier [98] (version 4.6-7). Most of the parameters were kept as default, but ntree was set to 1,000 to balance efficiency and accuracy in our high-dimensional data. We found the parameter settings for the RF classifier (including the number of trees) to be robust to different settings, so we did not estimate parameters in our classifier. The Gini index, which calculates the total decrease of node impurity (i.e., the relative entropy of the class proportions before and after the split) of a feature over all trees, was used to quantify the importance of each feature:

(3) $$ I(A)=1-\sum_{k=1}^{c}{p_{k}^{2}},  $$

where *k* represents the class and *p*_*k*_ is the proportion of sites belonging to class *k* in node *A*.

We used the SVM implementation in the e1071 package in R [99] with a radial basis function kernel. The parameters of the SVM were optimized by tenfold cross-validation using a grid search. The penalty constant *C* ranged from 2^−1^,2^1^,…,2^9^ and the parameter *γ* in the kernel function ranged from 2^−9^,2^−7^,…,2^1^. The parameter combination that had the best performance – *γ*=2^−7^ and *C*=2^3^ – was used to generate the results used in the comparisons.

For k-NN, we used the knn function in R, with the number of neighbors equal to the square root of the number of samples in the training set. For the logistic regression classifier, we used the logistic regression classifier implemented in the R base package with the function glm and family = ‘binomial’. We set the threshold for classification to $\hat {\beta }_{i,j} \geq 0.5$. For the naive Bayes classifier, we used the naiveBayes function in the R e1071 package.

### Features for prediction

A comprehensive list of 124 features were used in prediction (Additional file 1: Table S2). The neighbor features were obtained from data from the Methylation 450K Array. The position features, including gene coding region category, location in CGIs, and SNPs, were obtained from the Methylation 450K Array Annotation file. DNA recombination rate data were downloaded from HapMap (phaseII_B37, update date 19 January 2011) [55]. GC content data were downloaded from the raw data used to encode the gc5Base track on hg19 (update date 24 April 2009) from the UCSC Genome Browser [100,101]. iHSs were downloaded from the HGDP selection browser iHS data of *smoothedAmericas* (update date 12 February 2009) [57,102], and GERP constraint scores were downloaded from *SidowLab GERP++* tracks on hg19 [58,103].

For the CRE features, DHS sites data were obtained from the DNase-seq data for the GM12878 cell line produced by Crawford Lab at Duke University (UCSC accession [wgEncode:EH000534], submitted date 20 March 2009). Chromatin immunoprecipitation sequencing (ChIP-seq) data for 79 specific TFBSs were obtained from the narrow peak files from the GM12878 cell line that were available before June 2012 from the ENCODE website. Ten histone modifications were obtained from the peak files from the GM12878 cell line that were available before December 2013 from the ENCODE website and 15 chromatin states were obtained from the Broad ChromHMM data from the GM12878 cell line (UCSC accession [wgEncode:EH000033], submitted data 21 March 2011) [56].

Neighboring CpG site methylation status *τ* was encoded as methylated (*τ*=1) when the site has *β*≥0.5 and unmethylated (*τ*=0) when *β*<0.5. For continuous features, the feature value is the value of that feature at the genomic location of the CpG site; for binary features, the feature status indicates whether the CpG site is within that genomic feature or not. DHS sites were encoded as binary variables indicating a CpG site within a DHS site. TFBSs were included as binary variables indicating the presence of a co-localized ChIP-Seq peak. iHSs, GERP constraint scores and recombination rates were measured in terms of genomic regions. For GC content, we computed the proportion of G and C within a sequence window of 400 bp, as this feature was shown to be an important predictor in a previous study [41]. Among all 124 features, 122 of them (excluding *β* values of upstream and downstream neighboring CpG sites) were used for methylation status predictions, and all, excluding methylation status of upstream and downstream neighboring CpG sites *τ*, were used for methylation level predictions. When limiting prediction to specific regions, e.g., CGIs, we excluded those region-specific features from the data.

### Prediction evaluation

Our methylation predictions were at single-CpG-site resolution. For regional-specific methylation prediction, we grouped the CpG sites into either promoter, gene body, and intergenic region classes, or CGI, CGI shore and shelf, and non-CGI classes according to the Methylation 450K array annotation file, which was downloaded from the UCSC genome browser [51].

The classifier performance was assessed by a version of repeated random subsampling validation. Within a single individual, ten times we sampled 10,000 random CpG sites from across the genome into the training set, and we tested on all other held-out sites. The prediction performance for a single classifier was calculated by averaging the prediction performance statistics across each of the ten trained classifiers. We checked the performance with smaller training set of sizes 100, 1,000, 2,000, 5,000 and 10,000 sites in the same evaluation setup. In cross-sample analyses, we set the size of the training set to 10,000 randomly chosen CpG sites to balance computational performance and accuracy. We then evaluated the consistency of methylation pattern in different individuals by training the classifier using 10,000 randomly chosen CpG sites in one individual, and then using the trained classifier to predict all of the CpG sites for the remaining 99 individuals. In cross-gender analyses, we randomly chose 10,000 CpG sites from one randomly chosen female or male and tested on all CpG sites from another randomly chosen female or male. This was repeated ten times.

In cross-platform prediction and WGBS prediction, we sampled 10,000 randomly chosen CpG sites from 450K data or CpG sites categorized as 450K sites in WGBS data as training sets. We tested on 100,000 randomly chosen CpG sites that were categorized as 450K sites or non 450K sites in the WGBS data. The prediction performance for a single classifier was calculated by averaging the prediction performance statistics across each of the ten trained classifiers.

We quantified the accuracy of the results using the specificity (SP), sensitivity (recall) (SE), precision, accuracy (ACC), and Matthew’s correlation coefficient (MCC). Note that *truly significant* CpG sites are those that are methylated, and *truly null* CpG sites are those that are unmethylated in these data. These values were calculated as follows:

(4) $$ \text{SP}=\frac{\text{TN}}{\text{TN}+\text{FP}}  $$

(5) $$ \text{SE} =\frac{\text{TP}}{\text{TP}+\text{FN}}  $$

(6) $$ \text{Precision} =\frac{\text{TP}}{\text{TP}+\text{FP}}  $$

(7) $$ \text{Recall} =\frac{\text{TP}}{\text{TP}+\text{FN}}  $$

(8) $$ \text{ACC} =\frac{\text{TP}+\text{TN}}{\text{TP}+\text{FP}+\text{TN}+\text{FN}}  $$

(9) $$  \text{MCC} \,=\,\frac{\text{TP}\times \text{TN}-\text{FP}\!\times \! \text{FN}}{\sqrt{(\text{TP}\,+\,\text{FN})\!\times \!(\text{TP}\,+\,\text{FP})\!\times\!(\text{TN}\,+\,\text{FP})\!\times \!(\text{TN}\,+\,\text{FN})}},  $$

for true positives (TP), true negatives (TN), false positives (FP), and false negatives (FN) for a particular threshold. We computed ROC curves, precision–recall curves, AUC, and AUPR; AUC and AUPR reflect the overall prediction performance considering both type I (FPs) and type II errors (FNs) [39,104]. We used the ROCR package in R [105].

To estimate continuous methylation levels ($\hat {\beta }$), we used the classifier output of prediction probability from the RF classifier directly as an estimate of a specific *β*∈[0,1]. Prediction accuracy was evaluated using Pearson’s correlation coefficient and RMSE:

(10) $$ r_{x,y}=\frac{\sum_{j=1}^{p}(x_{j}-\bar{x})(y_{j}-\bar{y})}{(p-1)\cdot\sigma_{x}\cdot \sigma_{y}}  $$

(11) $$ \text{RMSE}_{x,y} = \sqrt{\frac{\sum_{j=1}^{p}(y_{j}-x_{j})^{2}}{p}},  $$

where *x*_*j*_,*y*_*j*_ are the experimental and predicted values for the *j*th CpG site, respectively, $\bar {x}$, $\bar {y}$ are the means of the experimental and predicted methylation levels, respectively, and *σ*_*x*_, *σ*_*y*_ are the empirical standard deviations of the experimental and predicted values, respectively.

### Availability of data and code

We have released the TwinsUK 450K Array data for 100 samples through GEO [GEO:GSE62992]. We have released the R code associated with the processing and analyses of these data on the Engelhardt Group website [106].

## Additional file

Additional file 1 **Supplementary Materials.** All supplemental tables and supplemental figures.

## Abbreviations

AUC: area under the receiver operating characteristic curve
AUPR: area under the precision–recall curve
bp: base pair
CGI: CpG island
ChIP-seq: chromatin immunoprecipitation sequencing
CRE: cis-regulatory element
DHS: DNAse I hypersensitive
GEO: Gene Expression Omnibus
GERP: genomic evolutionary rate profiling
iHS: integrated haplotype score
kb: kilobase
k-NN: *k*-nearest neighbors classifier
Mb: megabase
MCC: Matthew’s correlation coefficient
MED: mean squared Euclidean distance
PC: principal component
PCA: principal component analysis
QTL: quantitative trait locus
RF: random forest
RMSE: root-mean-square error
ROC: receiver operating characteristic
SNP: single nucleotide polymorphism
SVM: support vector machine
TF: transcription factor
TFBS: transcription factor binding site
WGBS: whole-genome bisulfite sequencing

## References

[1] Barrero MJ, Izpisúa Belmonte JC, Boué S (2010). Epigenetic mechanisms that regulate cell identity. Cell Stem Cell..

[2] Scarano MI, Strazzullo M, Matarazzo MR, D’Esposito M (2005). DNA methylation 40 years later: Its role in human health and disease. J Cell Physiol..

[3] Cedar H, Bergman Y (2012). Programming of DNA methylation patterns. Annu Rev Biochem..

[4] Kiefer JC (2007). Epigenetics in development. Dev Dyn..

[5] Tost J (2010). DNA methylation: an introduction to the biology and the disease-associated changes of a promising biomarker. Mol Biotechnol..

[6] Cedar H (1988). DNA methylation and gene activity. Cell..

[7] Jaenisch R, Bird A (2003). Epigenetic regulation of gene expression: how the genome integrates intrinsic and environmental signals. Nat Genet..

[8] Wolffe AP, Matzke MA (1999). Epigenetics: regulation through repression. Science.

[9] Rivenbark AG, Stolzenburg S, Beltran AS, Yuan X, Rots MG, Strahl BD, et al.Epigenetic reprogramming of cancer cells via targeted DNA methylation. Epigenetics Official J DNA Methylation Soc.2012;7. http://www.ncbi.nlm.nih.gov/pubmed/22419067. 10.4161/epi.19507 PMC3368819 22419067

[10] Das PM, Singal R (2004). DNA methylation and cancer. J Clin Oncol..

[11] Lienert F, Wirbelauer C, Som I, Dean A, Mohn F, Schübeler D (2011). Identification of genetic elements that autonomously determine DNA methylation states. Nat Genet..

[12] Jones PA. Functions of DNA methylation: islands, start sites, gene bodies and beyond. Nat Rev Genet. 201; 13:484–92. 10.1038/nrg3230 22641018

[13] Law JA, Jacobsen SE (2010). Establishing, maintaining and modifying DNA methylation patterns in plants and animals. Nat Rev Genet..

[14] Shen L, Kondo Y, Guo Y, Zhang J, Zhang L, Ahmed S (2007). Genome-wide profiling of DNA methylation reveals a class of normally methylated CpG island promoters. PLoS Genet..

[15] Larsen F, Gundersen G, Lopez R, Prydz H (1992). CpG islands as gene markers in the human genome. Genomics..

[16] Brandeis M, Frank D, Keshet I, Siegfried Z, Mendelsohn M, Nemes A (1994). Sp1 elements protect a CpG island from de novo methylation. Nature..

[17] Macleod D, Charlton J, Mullins J, Bird AP (1994). Sp1 sites in the mouse aprt gene promoter are required to prevent methylation of the CpG island. Genes Dev..

[18] Dickson J, Gowher H, Strogantsev R, Gaszner M, Hair A (2010). VEZF1 elements mediate protection from DNA methylation. PLoS Genet..

[19] Teschendorff AE, Menon U, Gentry-Maharaj A, Ramus SJ, Gayther SA, Apostolidou S (2009). An epigenetic signature in peripheral blood predicts active ovarian cancer. PLoS One..

[20] Deaton AM, Bird A (2011). CpG islands and the regulation of transcription. Genes Dev..

[21] Choy MK, Movassagh M, Goh HG, Bennett MR, Foo RSY, Down T a (2010). Genome-wide conserved consensus transcription factor binding motifs are hyper-methylated. BMC Genomics..

[22] Gebhard C, Benner C, Ehrich M, Schwarzfischer L, Schilling E (2010). General transcription factor binding at CpG islands in normal cells correlates with resistance to de novo DNA methylation in cancer cells. Cancer Res..

[23] Stirzaker C, Song JZ, Davidson B, Clark SJ (2004). Transcriptional gene silencing promotes DNA hypermethylation through a sequential change in chromatin modifications in cancer cells. Cancer Res..

[24] Valenzuela L, Kamakaka RT (2006). Chromatin insulators. Annu Rev Genet..

[25] Weber M, Hellmann I, Stadler MB, Ramos L, Pääbo S, Rebhan M (2007). Distribution, silencing potential and evolutionary impact of promoter DNA methylation in the human genome. Nat Genet..

[26] Meissner A, Mikkelsen TS, Gu H, Wernig M, Hanna J, Sivachenko A (2008). Genome-scale DNA methylation maps of pluripotent and differentiated cells. Nature..

[27] Hawkins RD, Hon GC, Lee LK, Ngo Q, Lister R, Pelizzola M (2010). Distinct epigenomic landscapes of pluripotent and lineage-committed human cells. Cell Stem Cell..

[28] Das R, Dimitrova N, Xuan Z, Haghighi F, Edwards JR, Rollins Ra (2006). Computational prediction of methylation status in human genomic sequences. Proc Natl Acad Sci U S A..

[29] Laird PW (2010). Principles and challenges of genomewide DNA methylation analysis. Nat Rev Genet..

[30] Laurent L, Wong E, Li G, Huynh T, Tsirigos A, Ong CT (2010). Dynamic changes in the human methylome during differentiation. Genome Res..

[31] Hon G, Antosiewicz-bourget J, Malley RO, Castanon R (2011). Hotspots of aberrant epigenomic reprogramming in human induced pluripotent stem cells. Nature..

[32] Lister R, Pelizzola M, Dowen RH, Hawkins RD, Hon G, Tonti-Filippini J, et al. (2009). Human DNA methylomes at base resolution show widespread epigenomic differences. Nature..

[33] Sandoval J, Heyn HA, Moran S, Serra-Musach J, Pujana MA, Bibikova M (2011). Validation of a DNA methylation microarray for 450,000 CpG sites in the human genome. Epigenetics Official J DNA Methylation Soc..

[34] Bibikova M, Barnes B, Tsan C, Ho V, Klotzle B, Le JM (2011). High density DNA methylation array with single CpG site resolution. Genomics..

[35] Bell JT, Pai AA, Pickrell JK, Gaffney DJ, Pique-Regi R, Degner JF (2011). DNA methylation patterns associate with genetic and gene expression variation in HapMap cell lines. Genome Biol..

[36] Eckhardt F, Lewin J, Cortese R, Rakyan VK, Attwood J, Burger M (2006). DNA methylation profiling of human chromosomes 6, 20 and 22. Nat Genet..

[37] Fernandez AF, Assenov Y, Martin-Subero JI, Balint B, Siebert R, Taniguchi H (2011). A DNA methylation fingerprint of 1628 human samples. Genome Res..

[38] Ma B, Wilker EH, Willis-Owen SAG, Byun HM, Wong KCC, Motta V (2014). Predicting DNA methylation level across human tissues. Nucleic Acids Res..

[39] Bhasin M, Zhang H, Reinherz EL, Reche PA (2005). Prediction of methylated CpGs in DNA sequences using a support vector machine. FEBS Lett..

[40] Bock C, Paulsen M, Tierling S, Mikeska T, Lengauer T, Walter J (2006). CpG island methylation in human lymphocytes is highly correlated with DNA sequence, repeats, and predicted DNA structure. PLoS Genet..

[41] Fang F, Fan S, Zhang X, Zhang MQ (2006). Predicting methylation status of CpG islands in the human brain. Bioinformatics (Oxford, England)..

[42] Kim S, Li M, Paik H, Nephew K, Shi H, Kramer R (2008). Predicting DNA methylation susceptibility using CpG flanking sequences. Pac Symp Biocomput..

[43] Fan S, Zhang MQ, Zhang X (2008). Histone methylation marks play important roles in predicting the methylation status of CpG islands. Biochem Biophys Res Commun..

[44] Lu L (2010). Predicting DNA methylation status using word composition. J Biomed Sci Eng..

[45] Zheng H, Wu H, Li J, Jiang SW (2013). CpGIMethPred: computational model for predicting methylation status of CpG islands in human genome. BMC Med Genomics..

[46] Previti C, Harari O, Zwir I, del Val C (2009). Profile analysis and prediction of tissue-specific CpG island methylation classes. BMC Bioinformatics..

[47] Maunakea AK, Nagarajan RP, Bilenky M, Ballinger TJ, D’Souza C, Fouse SD (2010). Conserved role of intragenic DNA methylation in regulating alternative promoters. Nature..

[48] Zhou X, Li Z, Dai Z, Zou X (2012). Prediction of methylation CpGs and their methylation degrees in human DNA sequences. Comput Biol Med..

[49] Siepel A, Bejerano G, Pedersen JS, Hinrichs AS, Hou M, Rosenbloom K (2005). Evolutionarily conserved elements in vertebrate, insect, worm, and yeast genomes. Genome Res..

[50] Heyn H, Carmona FJ, Gomez A, Ferreira HJ, Bell JT, Sayols S (2013). DNA methylation profiling in breast cancer discordant identical twins identifies DOK7 as novel epigenetic biomarker. Carcinogenesis..

[51] Kent WJ, Sugnet CW, Furey TS, Roskin KM, Pringle TH, Zahler a M (2002). The human genome browser at UCSC. Genome Res..

[52] Durbin RM, Altshuler DL, Abecasis GR, Bentley DR, Chakravarti A, Clark AG (2010). A map of human genome variation from population-scale sequencing. Nature..

[53] Keene MA, Corces V, Lowenhaupt K, Elgin SC (1981). DNase I hypersensitive sites in *Drosophila* chromatin occur at the 5^′^ ends of regions of transcription. Proc Natl Acad Sci U S A..

[54] Bernat JA, Crawford GE, Ogurtsov AY, Collins FS, Ginsburg D, Kondrashov AS (2006). Distant conserved sequences flanking endothelial-specific promoters contain tissue-specific DNase-hypersensitive sites and over-represented motifs. Hum Mol Genet..

[55] International HapMap Consortium (2005). A haplotype map of the human genome. Nature..

[56] Good PJ, Guyer MS, Kamholz S, Liefer L, Wetterstrand K, Kampa D (2004). The ENCODE (ENCyclopedia Of DNA Elements) Project. Science..

[57] Voight BF, Kudaravalli S, Wen X, Pritchard JK (2006). A map of recent positive selection in the human genome. PLoS Biol..

[58] Davydov EV, Goode DL, Sirota M, Cooper GM, Sidow A, Batzoglou S (2010). Identifying a high fraction of the human genome to be under selective constraint using GERP++. PLoS Comput Biol..

[59] Ziller MJ, Gu H, Müller F, Donaghey J, Tsai LTY, Kohlbacher O, et al. Charting a dynamic DNA methylation landscape of the human genome. Nature. 2013,:1–5. http://www.nature.com/doifinder/10.1038/nature12433. 10.1038/nature12433 PMC3821869 23925113

[60] Hodges E, Molaro A, Dos Santos CO, Thekkat P, Song Q, Uren PJ (2011). Directional DNA methylation changes and complex intermediate states accompany lineage specificity in the adult hematopoietic compartment. Mol Cell..

[61] He H, Garcia E (2009). Learning from imbalanced data. IEEE Trans Knowl Data Eng..

[62] Irizarry RA, Ladd-Acosta C, Wen B, Wu Z, Montano C, Onyango P (2009). Genome-wide methylation analysis of human colon cancer reveals similar hypo- and hypermethylation at conserved tissue-specific CpG island shores. Nat Genet..

[63] Doi A, Park IH, Wen B, Murakami P, Aryee MJ, Irizarry R (2009). Differential methylation of tissue- and cancer-specific CpG island shores distinguishes human induced pluripotent stem cells, embryonic stem cells and fibroblasts. Nat Genet..

[64] Tsumagari K, Baribault C, Terragni J, Varley KE, Gertz J, Pradhan S (2013). Early de novo DNA methylation and prolonged demethylation in the muscle lineage. Epigenetics : Official J DNA Methylation Soc..

[65] Hogart A, Lichtenberg J, Ajay SS, Anderson S, Intramural NIH, Margulies EH (2012). Genome-wide DNA methylation profiles in hematopoietic stem and progenitor cells reveal overrepresentation of ETS transcription factor binding sites. Genome Res..

[66] Chuang LSH, Ito Y (2010). RUNX3 is multifunctional in carcinogenesis of multiple solid tumors. Oncogene..

[67] Li QL, Ito K, Sakakura C, Fukamachi H, Inoue KI, Chi XZ (2002). Causal relationship between the loss of RUNX3 expression and gastric cancer. Cell..

[68] Kim WJ, Kim EJ, Jeong P, Quan C, Kim J, Li QL (2005). RUNX3 inactivation by point mutations and aberrant DNA methylation in bladder tumors. Cancer Res..

[69] Lau QC, Raja E, Salto-Tellez M, Liu Q, Ito K, Inoue M (2006). RUNX3 is frequently inactivated by dual mechanisms of protein mislocalization and promoter hypermethylation in breast cancer. Cancer Res..

[70] Sato K, Tomizawa Y, Iijima H, Saito R, Ishizuka T, Nakajima T (2006). Epigenetic inactivation of the RUNX3 gene in lung cancer. Oncol Rep..

[71] Weisenberger D, D Siegmund K, Campan M, Young J, Long T, Faasse M (2006). CpG island methylator phenotype underlies sporadic microsatellite instability and is tightly associated with BRAF mutation in colorectal cancer. Nat Genet..

[72] Lázcoz P, Muñoz J, Nistal M, Pestaña A, Encío IJ, Castresana JS (2007). Loss of heterozygosity and microsatellite instability on chromosome arm 10q in neuroblastoma. Cancer Genet Cytogenet..

[73] Song J, Ugai H, Kanazawa I, Sun K, Yokoyama KK (2001). Independent repression of a GC-rich housekeeping gene by Sp1 and MAZ involves the same cis-elements. J Biol Chem..

[74] Song J, Ugai H, Nakata-Tsutsui H, Kishikawa S, Suzuki E, Murata T (2003). Transcriptional regulation by zinc-finger proteins Sp1 and MAZ involves interactions with the same cis-elements. Int J Mol Med..

[75] Baron B (2012). Breaking the silence: the interplay between transcription factors and DNA methylation. Methylation – from DNA, RNA and histones to diseases and treatment.

[76] Guccione E, Martinato F, Finocchiaro G, Luzi L, Tizzoni L (2006). Myc-binding-site recognition in the human genome is determined by chromatin context. Nat Cell Biol.

[77] Toyota M, Suzuki H (2010). Epigenetic drivers of genetic alterations. Adv Genet..

[78] Esteller M, Toyota M, Sanchez-Cespedes M, Capella G, Peinado MA, Watkins DN (2000). Inactivation of the DNA repair gene O6-methylguanine-DNA methyltransferase by promoter hypermethylation is associated with G to A mutations in K-ras in colorectal tumorigenesis. Cancer Res..

[79] Yang J, Ferreira T, Medland SE, Madden PAF, Morris A P, Heath AC, et al. (2012). Conditional and joint multiple-SNP analysis of GWAS summary statistics identifies additional variants influencing complex traits. Nat Genet..

[80] Mangravite LM, Engelhardt BE, Medina MW, Smith JD, Brown CD, Chasman DI (2013). A statin-dependent QTL for GATM expression is associated with statin-induced myopathy. Nature..

[81] Lokk K, Modhukur V, Rajashekar B, Märtens K, Mägi R, Kolde R (2014). DNA methylome profiling of human tissues identifies global and tissue-specific methylation patterns. Genome Biol..

[82] Jaffe AE, Irizarry RA (2014). Accounting for cellular heterogeneity is critical in epigenome-wide association studies. Genome Biol..

[83] Howie BN, Donnelly P, Marchini J (2009). A flexible and accurate genotype imputation method for the next generation of genome-wide association studies. PLoS Genet..

[84] Howie B, Stephens M, Marchini J, Abecasis GR, Fuchsberger C (2012). Fast and accurate genotype imputation in genome-wide association studies through pre-phasing. Nat Genet..

[85] Zhu Q, Ge D, Maia JM, Zhu M, Petrovski S, Dickson SP (2011). A genome-wide comparison of the functional properties of rare and common genetic variants in humans. Am J Hum Genet..

[86] McClellan J, King MC (2010). Genetic heterogeneity in human disease. Cell.

[87] Zou J, Lippert C, Heckerman D, Aryee M, Listgarten J (2014). Epigenome-wide association studies without the need for cell-type composition. Nat Methods.

[88] Gibbs JR, Van Der Brug, Hernandez DG, Traynor BJ, Nalls MA, Lai SL (2010). Abundant quantitative trait loci exist for DNA methylation and gene expression in human brain. PLoS Genet..

[89] Zhang D, Cheng L, Badner JA, Chen C, Chen Q, Luo W (2010). Genetic control of individual differences in gene-specific methylation in human brain. Am J Hum Genet..

[90] Degner JF, Pique-Regi R, Veyrieras JB, Gaffney DJ, Pickrell JK, Pai Aa (2012). DNase I sensitivity QTLs are a major determinant of human expression variation. Nature..

[91] Pai AA, Cain CE, Mizrahi-Man O, De Leon S, Lewellen N, Veyrieras JB, et al. (2012). The contribution of RNA decay quantitative trait loci to inter-individual variation in steady-state gene expression levels. PLoS Genet..

[92] Gaffney DJ, Veyrieras JB, Degner JF, Pique-Regi R, Pai AA, Crawford GE (2012). Dissecting the regulatory architecture of gene expression QTLs. Genome Biol..

[93] Moayyeri A, Hammond CJ, Valdes AM, Spector TD. Cohort profile: TwinsUK and healthy ageing twin study. Int J Epidemiol. 2012. http://www.ncbi.nlm.nih.gov/pubmed/22253318. 10.1093/ije/dyr207 PMC3600616 22253318

[94] Rechache NS, Wang Y, Stevenson HS, Killian JK, Edelman DC, Merino M (2012). DNA methylation profiling identifies global methylation differences and markers of adrenocortical tumors. J Clin Endocrinol Metab..

[95] R project. http://www.r-project.org/.

[96] Gabriel KR, Odoroff CL (1990). Biplots in biomedical research. Stat Med..

[97] Bioconductor open source software for bioinformatics. http://www.bioconductor.org/.

[98] Liaw A, Wiener M (2002). Classification and regression by randomForest. R News.

[99] Meyer D, Dimitriadou E, Hornik K, Weingessel A, Leisch F. Misc functions of the Department of Statistics (e1071). 2012. http://cran.r-project.org/package=e1071.

[100] Golden Path track of the University of Santa Cruz Genome Browser. http://hgdownload.cse.ucsc.edu/goldenPath/hg19/gc5Base/.

[101] Meyer LR, Zweig AS, Hinrichs AS, Karolchik D, Kuhn RM, Wong M (2013). The UCSC genome browser database: extensions and updates 2013. Nucleic Acids Res.

[102] Integrated Haplotype Scores from the University of Chicago. http://hgdp.uchicago.edu/data/iHS/.

[103] Genomic Evolutionary Rate Profiling from the Sidow Lab at Stanford University. http://mendel.stanford.edu/SidowLab/downloads/gerp/.

[104] Fogarty J, Baker RS, Hudson SE. Case studies in the use of ROC curve analysis for sensor-based estimates in human computer interaction. In: Inkpen K, Van De Panne M, editors. GI 05 Proceedings of Graphics Interface 2005, ACM International Conference Proceeding Series. Canadian Human-Computer Communications Society, Canadian Human-Computer Communications Society: 2005. p. 129–36. http://www.cs.cmu.edu/afs/cs.cmu.edu/misc/mosaic/common/omega/Web/People/jfogarty/publications/gi2005.pdf.

[105] Sing T, Sander O, Beerenwinkel N, Lengauer T (2005). ROCR: visualizing classifier performance in R. Bioinformatics (Oxford, England).

[106] Open-source software from the Engelhardt Group at Princeton University. http://www.cs.princeton.edu/~bee/software.html.

[107] Ernst J, Kellis M (2012). ChromHMM: automating chromatin-state discovery and characterization. Nat. Methods..

