# Supplementary material for: Predicting genome-wide DNA methylation using methylation marks, genomic position, and DNA regulatory elements
Source: Genome Biol. 2015 Jan 24;16(1):14. doi: 10.1186/s13059-015-0581-9 (PMC4389802; doi:10.1186/s13059-015-0581-9)
Supplement: Supplementary file 1 — Supplementary Materials. All supplemental tables and supplemental figures. [file 13059_2015_581_MOESM1_ESM.pdf]

Table 1: **Related work on DNA methylation prediction.** MethDB is a database of measurements of DNA methylation from variety of studies and methods [1], which is regularly updated. HEP: Human Genome Project, which contains about 1.9 million CpG methylation values from chromosomes 6, 20 and 22 from 12 cell types across 43 samples.

	Data set	Classifier	Features	Training set	Prediction	Best or overall performance
Bhasin *et al.* 2005	MethDB with methylation value of 5000 DNA fragments	SVM	DNA composition	Methylation status of DNA fragment with window size of 9-89bp	Methylation Status	ACC: 75%, AUC: 0.82, MCC 0.504
Bock *et al.* 2006	Measurements of CGI methylation on human Chromosome 21 (Yamada et al. 2004) with methylation status of 149 CGIs	SVM	DNA composition, predicted DNA structure, repeat element, TFBSs, evolutionary conservation elements, number of SNPs	CGIs methylation status	Methylation status	ACC: 91.5%
Das *et al.* 2006	Human brain data (Rollin et al. 2006) with methylation status of 5000 DNA domains	SVM (K-means clustering, linear discriminant analysis, logistic regression)	DNA composition	Methylation status of DNA fragments with window size of 800 bp	Methylation status	ACC: Overall: 86%; CGIs: 96.5%; non-CGIs: 84%
Fang *et al.* 2006	Human brain data (Rollin et al. 2006) with methylation status of 4000 DNA domains	SVM	GC content, Alu element, TFBSs	Methylation status of CG-rich regions with window size of 100-400 bp	Methylation status	ACC: 84.52% CC: 0.686
Kim *et al.* 2007	Sequencing data on 25 gene-related CGIs	Naive Bayes (SMO, Multilayer Perception, Instance Based Classifier)	DNA composition	CGI methylation status of DNA fragments with window size of 30bp	Methylation status	ACC: over 75%
Fan *et al.* 2008	HEP (Eckhardt et al. 2006) with methylated status of 500 DNA fragments of T cell	SVM	DNA composition, histone methylation marks	CGIs methylation status	Methylation status	ACC: 89.94%
Previti *et al.* 2009	HEP with methylated status of 400-500 DNA fragments in 12 cell types	Decision tree (SVM)	GC content, repetitive sequences, evolutionary conservation, DNA structure	CGI methylation status	Methylation status	CC: 0.775 ACC: 91.67%
Lu *et al.* 2010	HEP with methylated status of 3500 DNA fragments of CD4 lymphocytes	Word composition based encoding method	DNA composition	Methylation status of DNA fragments with window size of 1000 bp	Methylation status	ACC: 77.45%
Zhou *et al.* 2012	MethDB with methylation value of 400 human gene fragments	SVM	DNA composition	Methylation level of DNA fragments with window size of 19-129 bp	Methylation status and levels	Methylation status prediction: ACC: 0.82, MCC: 0.64; methylation level prediction: R: 0.82, RMSE: 0.2042
Zheng *et al.* 2013	HEP with methylated status of 400-500 DNA fragments from 12 cell types	SVM	DNA composition, Conserved TFBSs, DNA structure, functional role of nearby genes and histone marks	CGIs methylation status	Methylation status	ACC: ranges from 72% to 95% with different feature combinations in different cell types
Ma *et al.* 2014	Predicting DNA methylation level across human tissues	SVM with RBF kernel	Methylation levels in one cell type at same CpG site	Methylation levels of CpG sites in different tissues	Methylation levels	Methylation level prediction: r^2: 0.89-0.95

Table 2: **List of features used in prediction.** Columns include the category of features, the source of the data, the name of the features, and the feature encoding (either binary or numeric).

Category	Source	Feature Name	Data Type
Neighbors	Methylation 450K data	Upstream neighboring CpG site methylation status & level	binary & numeric
		Downstream neighboring CpG site methylation status & level	binary & numeric
	Distance features	Upstream neighboring distance	numeric
		Downstream neighboring distance	numeric
Genomic Position	Methylation 450K annotation	SNPs present within probe > 10bp from query site	binary
		SNPs present within probe < 10bp from query site	binary
		Presence in promoter	binary
		Presence in gene body	binary
		Presence in intergenic region	binary
		Presence in CpG island	binary
		Presence in CGI shore	binary
		Presence in CGI shelf	binary
		Presence in non-CGI region	binary
DNA sequence property	Genome Evolutionary Rate Profiling (GERP)	Constraint Score	numeric
	hgdp selection browser	Integrated Haplotype Score (iHS)	numeric
	HapMap	Recombination Rate	numeric
	ENCODE	GC Content	numeric
Cis-regulatory elements	ENCODE	DNAse I hypersensitive sites	binary
		CTCF	binary
		Cfos	binary
		E2F4	binary
		EBF1	binary
		ELK1	binary
		GCN5	binary
		IKZF1	binary
		IRF3	binary
		Jund	binary
		MAZ	binary
		Max	binary
		Mxi1	binary
		NFE2	binary
		NFYA	binary
		NFYB	binary
		Nfkb	binary
		Nrf1	binary
		P300	binary
		Pol2	binary
		Pol2_S2	binary
		Pol3	binary
		RFX5	binary
		Rad21	binary
		SIN3A	binary
		SMC3	binary
		SPT20	binary
		STAT1	binary
		STAT3	binary
		TBLR1	binary
		TBP	binary
		Tr4	binary
		USF2	binary
		WHIP	binary
		Yy1	binary

ZNF143	binary
ZNF274	binary
Zzz3	binary
Atf2	binary
Atf3	binary
Batf	binary
Bclaf1	binary
Bcl11a	binary
Bcl3	binary
Egr1	binary
Elf1	binary
Ets1	binary
Foxm1	binary
Gabp	binary
Irf4	binary
Mef2a	binary
Mef2c	binary
Mta3	binary
Nfatc1	binary
Nfic	binary
Nrsf	binary
Pax5	binary
Pbx3	binary
Pml	binary
Pol24h8	binary
Pou2f2	binary
Pu1	binary
RFX5	binary
Runx3	binary
Rxra	binary
Six5	binary
Sp1	binary
Srf	binary
Stat5a	binary
Taf1	binary
Tcf12	binary
Tcf3	binary
Usf1	binary
Zbtb33	binary
Zeb1	binary
c.Myc	binary
H3K4me1	binary
H3K04me3	binary
H3K4me2	binary
H3K9ac	binary
H3K9me3	binary
H3K27ac	binary
H3K27me3	binary
H3K36me3	binary
H3K79me2	binary
H4k20me1	binary
Active promoter	binary
Weak promoter	binary
Inactive promoter	binary
Strong enhancer	binary
Strong enhancer2	binary
Weak enhancer	binary
Weak enhancer2	binary
Insulator	binary
Transcriptional transition	binary
Transcriptional elongation	binary
Weak transcribed	binary
Polycomb	binary
Heterochromatin	binary

	Repetitive	binary
	CNV	binary

Table 3: **Region specific methylation prediction.** Num_sites: Number of CpG sites; Num_sites_1k: Number of sites with both neighbors within 1 kb; Methy%: Percentage of n corresponding regions with neighboring CpG site within 1 kb distance; Num_CV: Number of random subsamples.

Region	Num_sites	Num_sites_1k	Methy%	CV	Accuracy (%)	AUC	Precision (%)	Recall (%)	MCC	R	RMSE
Promoter	157,468	108,063	0.2070	10	94.98	0.9836	89.06	86.41	0.8457	0.9231	0.1346
Gene body	117,424	35,072	0.6369	3	93.45	0.9741	94.13	95.60	0.8581	0.9247	0.1681
Intergenic	91,177	25,694	0.3960	2	93.05	0.9738	91.23	90.59	0.8521	0.9176	0.1689
CGI	110,612	66,533	0.0973	6	98.32	0.9931	93.94	88.50	0.9025	0.9433	0.0926
CGI shore	89,989	28,232	0.4210	2	89.83	0.9584	88.46	87.15	0.7906	0.8892	0.1814
CGI shelf	36,658	4,736	0.7445	4	89.79	0.9514	89.88	97.54	0.7280	0.8779	0.1871
non-CGI	141,418	34,657	0.7149	3	91.93	0.9489	92.32	96.69	0.7991	0.8914	0.1910
All	378,677	189,735	0.3228	10	91.85	0.9624	90.61	95.06	0.8357	0.9016	0.1919

Table 4: **Number of occurrences and methylation levels of binary features.** Columns are Feature name, Feature counts (or the number of CpG sites that co-occur with these features in our data), and percentage of methylation CpG sites (or the proportion of CpG sites that co-occur with these features that are methylated).

Feature Name	Feature counts	Percentage of methylated CpG sites
Upstream neighboring CpG site methylation status	208536	0.841
Downstream neighboring CpG site methylation status	208535	0.841
SNPs present within probe $>$ 10bp from query site	45374	0.578
SNPs present within probe $<$ 10bp from query site	26557	0.64
DHS site	121516	0.111
Presence in CGI	110612	0.161
Presence in CGI Shore	89989	0.468
Presence in CGI Shelf	36658	0.864
Presence in non-CGI	141418	0.819
Presence in Promoter	157468	0.314
Presence in Gene body	117424	0.729
Presence in Intergenic region	91177	0.673
Atf2	28854	0.067
Atf3	7280	0.017
BHLHE40	70342	0.05
BRCA1	18594	0.023
Batf	14022	0.162
Bcl11a	7539	0.163
Bcl3	2759	0.356
Bclaf1	33688	0.05
CHD1	54108	0.041
CHD2	72260	0.042
COREST	58915	0.042
CTCF	48396	0.071
Cfos	5154	0.004
E2F4	17883	0.011
EBF1	56467	0.089
ELK1	44385	0.021
Egr1	5257	0.016
Elf1	89501	0.048
Ets1	14155	0.01
Foxm1	52346	0.043
GCN5	4176	0.023
Gabp	15904	0.007
IKZF1	8503	0.208
IRF3	11087	0.023
Irf4	14074	0.098
Jund	155	0.468
MAZ	83459	0.035
Max	77542	0.037
Mef2a	5499	0.12
Mef2c	4813	0.125
Mta3	88190	0.101
Mxi1	80851	0.042
NFE2	6491	0.039
NFYA	10329	0.018
NFYB	28315	0.042
Nfatc1	38120	0.089

Nfic	20773	0.084
Nfkb	4235	0.183
Nrf1	42227	0.018
Nrsf	4514	0.023
P300	39077	0.043
Pax5	42760	0.051
Pbx3	12800	0.044
Pml	81813	0.07
Pol2	77520	0.086
Pol24h8	68843	0.124
Pol2_S2	77669	0.14
Pol3	255	0.006
Pou2f2	55632	0.055
Pu1	17400	0.082
RFX5	36423	0.025
Rad21	11054	0.1
Runx3	85861	0.067
Rxra	1410	0.029
SIN3A	55349	0.014
SMC3	36131	0.08
SPT20	1867	0.112
STAT1	58324	0.03
STAT3	15220	0.083
Six5	7402	0.025
Sp1	51817	0.03
Srf	2987	0.043
Stat5a	65821	0.067
TBLR1	59257	0.045
TBP	50953	0.041
Taf1	52199	0.015
Tcf12	11512	0.083
Tcf3	17033	0.051
Tr4	2589	0.061
USF2	34220	0.048
Usf1	7466	0.034
WHIP	57505	0.092
Yy1	1827	0.044
ZNF143	75729	0.043
ZNF274	194	0.696
Zbtb33	5558	0.048
Zeb1	37222	0.033
Zzz3	838	0.049
c.Myc	11989	0.019
H3K4me1	132850	0.369
H3K4me3	155476	0.235
H3K4me2	111551	0.210
H3K9ac	166816	0.52
H3K9me3	133511	0.51
H3K27ac	155274	0.225
H3K27me3	118352	0.325
H3K36me3	122475	0.191
H3K79me2	73603	0.507
H4K20me1	188613	0.524
Active promoter	70341	0.041
Weak promoter	25332	0.236

Inactive promoter	11843	0.43
Strong enhancer	11843	0.43
Strong enhancer2	3720	0.714
Weak enhancer	13960	0.375
Weak enhancer2	8930	0.784
Insulator	6438	0.367
Transcriptional transition	4255	0.876
Transcriptional elongation	18777	0.956
Weak transcribed	35266	0.918
Polycomb	41145	0.36
Heterochromatin	121981	0.881
Repetitive	614	0.480
CNV	210	0.567

Table 5: **Gini importance scores for all features.** Gini_all: Gini scores for prediction in any genomic regions; Gini_promoter: Gini scores for prediction in promoter regions; Gini_CGI: Gini scores for prediction in CGIs.

Feature Name	Gini_all	Gini_promoter	Gini_CGI
Upstream neighbor CpG site methylation status	541.552	534.886	399.055
Downstream neighbor CpG site methylation status	531.864	535.967	402.254
DHS site	273.547	131.399	44.035
Upstream neighbor CpG site distance	241.021	77.379	22.678
Downstream neighbor CpG site distance	237.204	78.518	23.936
GC content	168.279	118.312	32.919
Elf1	162.508	77.157	19.12
H3K27ac	149.77	59.323	43.451
Within CGI	148.856	121.591	NA
MAZ	137.958	72.734	16.621
H3K4me3	110.184	50.229	36.9
Recombination rate	104.877	59.93	22.709
Mxi1	99.106	52.39	8.424
Runx3	94.866	37.644	7.971
Max	82.612	39.422	9.318
Heterochromatin	79.684	106.136	77.419
ZNF143	76.93	37.272	8.703
Polycomb	63.44	27.453	34.14
Active promoter	62.11	24.761	5.7
Pml	61.002	27.392	5.936
H3K9ac	55.64	33.347	50.589
Presence of non-CGI	54.75	94.865	NA
H3K36me3	53.501	22.92	13.819
iHS	47.941	28.896	10.067
Mta3	47.802	18.679	4.93
CHD2	44.864	27.873	5.476
Constraint score	42.809	29.204	13.145
BHLHE40	35.904	20.189	4.537
H3K9me3	32.004	18.461	15.002
Presence of promoter	31.598	NA	36.049
Pol2	29.515	17.188	3.097
Weak transcribed	25.533	25.837	41.617
Presence of CGI shelf	25.461	6.503	NA
Presence of CGI shore	24.028	20.577	NA
H3K4me2	23.788	12.885	6.843
Presence of gene	21.74	NA	39.436
SIN3A	21.18	12.887	2.066
H4K20me1	21.122	13.009	5.988
CTCF	19.86	8.4	2.733
Stat5a	19.513	8.783	2.268
STAT1	18.935	10.579	1.749
H3K79me2	18.573	11.452	5.647
H3K27me3	18.487	12.541	4.745
H3K04me1	18.311	11.599	5.42
Presence in intergenic region	15.118	NA	4.743
TBLR1	14.654	7.967	1.551
COREST	14.605	8.455	1.497
WHIP	12.743	6.98	0.507
SMC3	12.541	4.34	1.252

SNPs present within probe > 10bp from query site	11.814	6.986	2.629
Taf1	11.667	8.857	1.414
CHD1	11.445	6.645	1.178
Pol2_S2	10.963	10.742	2.869
SNPs present within probe < 10bp from query site	10.129	5.429	2.292
EBF1	10.062	6.626	1.02
Weak enhancer	9.716	6.95	3.011
Weak promoter	9.302	6.965	2.074
Transcriptional elongation	8.955	3.349	14.994
Sp1	8.231	8.698	2.218
Weak enhancer2	8.018	6.15	1.685
Pu1	7.836	2.423	0.327
Pol24h8	7.65	6.756	1.829
Inactive promoter	7.448	6.177	1.147
Pou2f2	7.413	6.137	1.42
Strong enhancer	7.401	6.207	1.184
Foxm1	6.941	5.752	0.924
Insulator	6.57	2.888	2.127
Rad21	6.496	1.607	0.399
TBP	5.239	5.528	0.605
Strong enhancer2	4.939	2.177	0.486
P300	4.913	3.742	0.424
ELK1	4.727	3.497	0.434
Zeb1	4.655	4.064	1.558
Pax5	4.565	2.944	1.251
Transcriptional transition	3.65	1.876	1.74
NFYB	3.617	3.331	0.643
IKZF1	3.583	1.85	0.07
Batf	3.542	1.382	0.091
USF2	3.48	2.833	0.502
Nfatc1	3.422	3.083	0.725
Nrf1	3.32	3.734	0.905
RFX5	2.818	2.333	0.321
Atf2	2.431	1.831	0.184
Bclaf1	2.333	2.099	0.366
Nfic	2.162	1.299	0.076
STAT3	2.023	1.503	0.053
Nfkb	1.94	1.316	0.036
Irf4	1.91	1.25	0.188
Tcf12	1.827	0.999	0.355
Bcl11a	1.813	0.951	0.224
Pbx3	1.631	0.801	0.123
Bcl3	1.458	0.569	0.651
Mef2a	1.365	0.725	0.059
Tcf3	1.328	0.679	0.409
Repetitive	1.23	0.743	0.426
BRCA1	1.218	0.829	0.213
Nrsf	1.147	0.783	0.288
Mef2c	1.109	0.663	0.027
Usf1	1.047	0.775	0.314
c.Myc	0.764	0.697	0.299
Zbtb33	0.631	0.402	0.095
IRF3	0.583	0.592	0.024
Six5	0.564	0.778	0.029
E2F4	0.546	0.595	0.098

Gabp	0.529	0.616	0.208
NFE2	0.515	0.426	0.042
NFYA	0.512	0.404	0.045
Ets1	0.418	0.635	0.116
Atf3	0.418	0.525	0.034
Tr4	0.404	0.431	0.005
GCN5	0.334	0.314	0.045
Yy1	0.31	0.432	0.026
SPT20	0.307	0.326	0.044
Srf	0.282	0.181	0.103
CNV	0.256	0.106	0.21
Egr1	0.232	0.35	0.122
Jund	0.178	0.177	0
Zzz3	0.145	0.246	0.017
ZNF274	0.13	0.024	0
Rxra	0.088	0.012	0.027
Cfos	0.057	0.091	0.011
Pol3	0.006	0.002	0

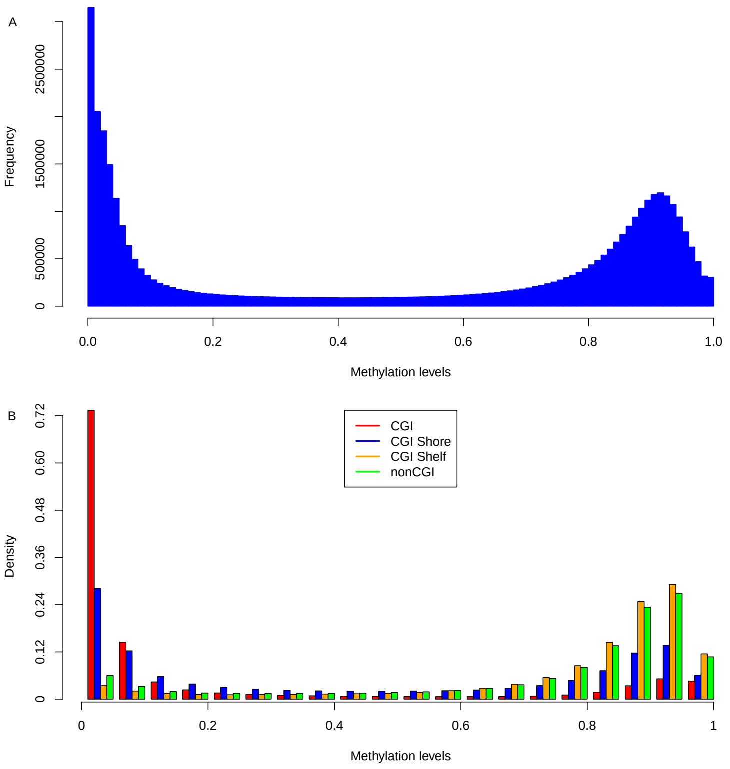

Figure 1: **Distribution of DNA methylation levels at CpG sites across autosomal chromosomes.** Methylation levels across 100 individuals at CpG sites assayed on the 450K array. Panel A: Distribution of DNA methylation values across all CpG sites. Panel B: Distribution of DNA methylation values for CpG sites within CGIs, CGI shores, CGI shelves, and non-CGI regions.

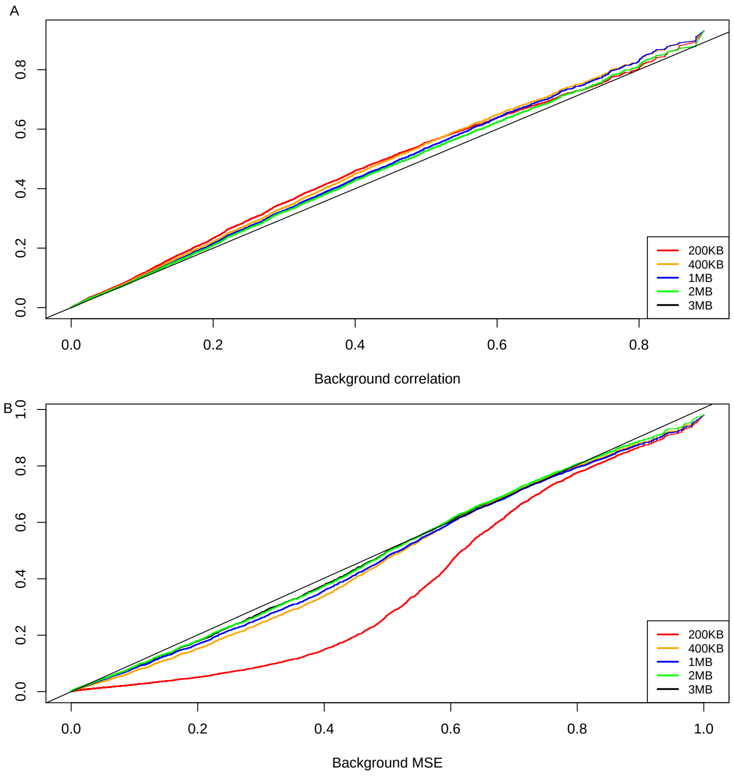

Figure 2: **QQ-plot of correlation and MED of methylation levels of arbitrary pairs of CpG sites.** Plot includes $10,000$ CpG sites for each panel, for background values and correlation/MED values. Different colors represent different genomic distances between pairs of CpG sites. Panel A: Quantile-quantile plot of correlation of methylation levels of pairs of CpG sites within a certain distance (y-axis) versus arbitrary pairs of CpG sites (x-axis). Panel B: Quantile-quantile plot of MED of methylation levels of pairs of CpG sites within a certain distance (y-axis) versus arbitrary pairs of CpG sites (x-axis).

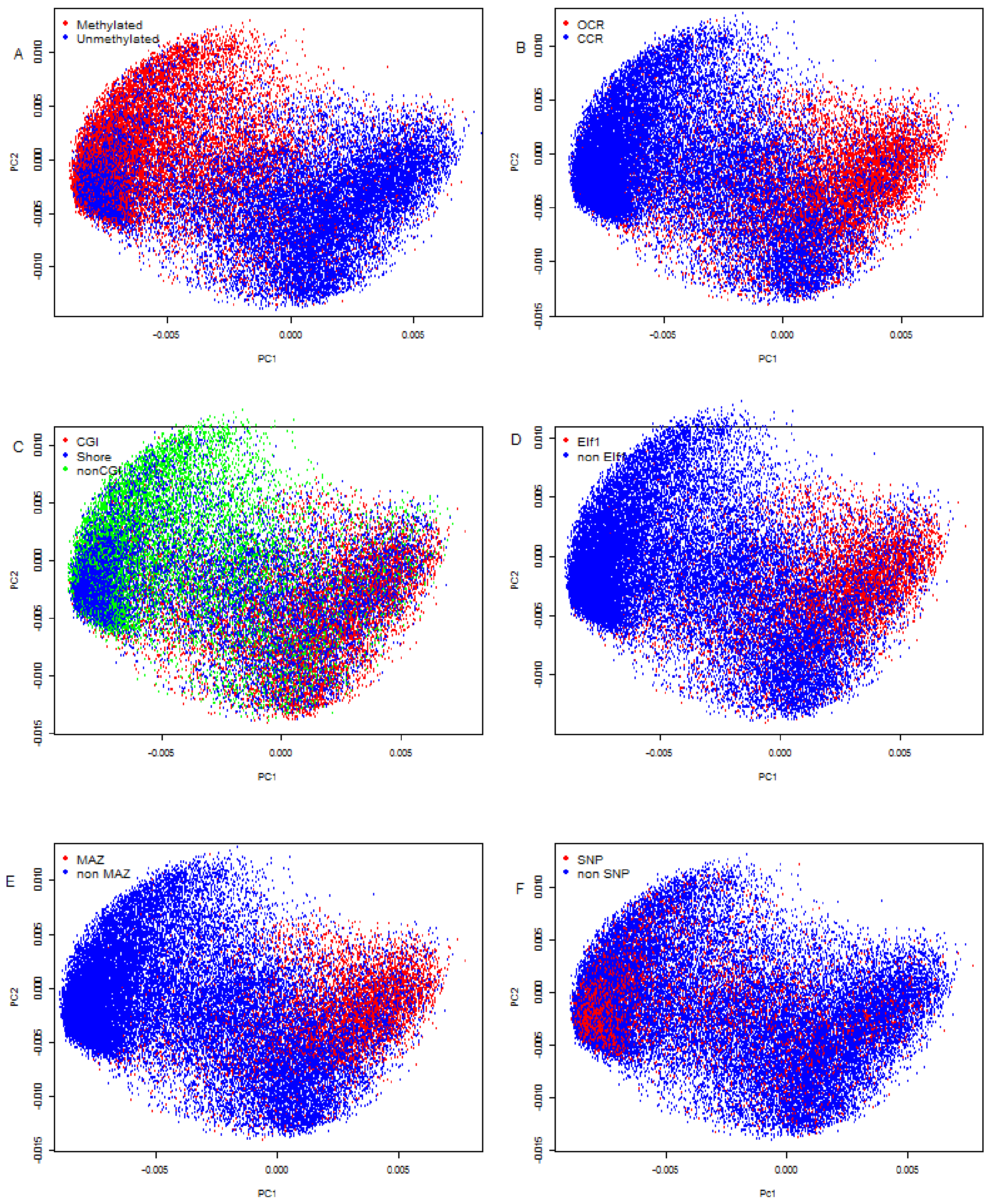

Figure 3: **PCA analysis of DNA methylation values.** PCA was performed on methylation levels of 10% of CpG sites. The first two principle component (PC1 and PC2) are plotted; colors correspond to the categories of features in the label.

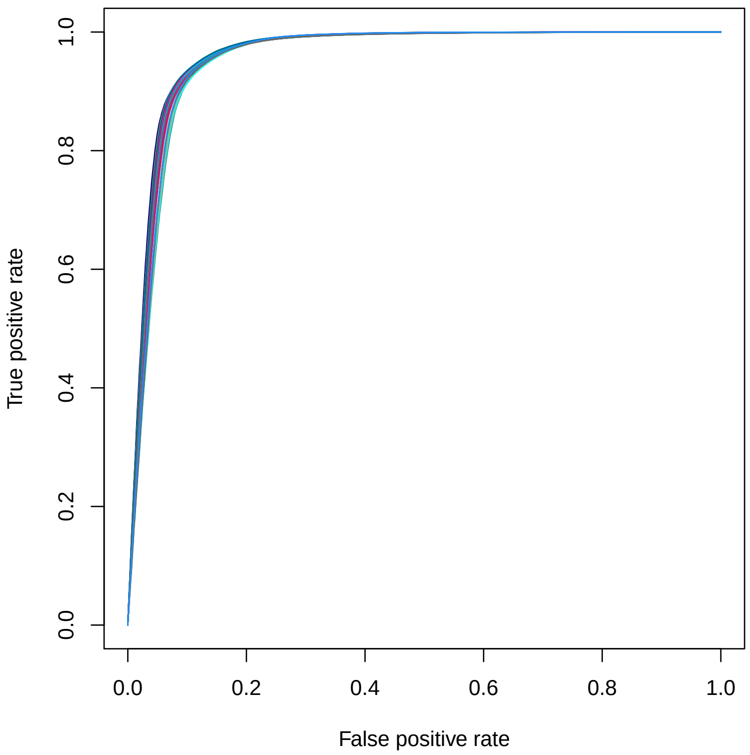

Figure 4: **ROC curves for methylation status prediction.** Each line represents the ROC curve for prediction of CpG sites, training and testing the classifier on data from one individual.

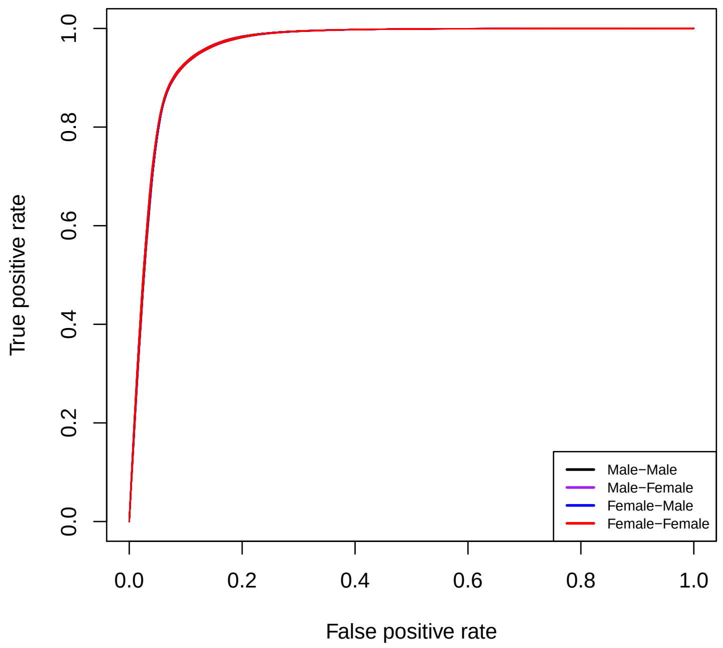

Figure 5: **ROC curves for methylation status prediction across sex.** Each line represents the ROC curve averaged over ten iterations of repeated random subsampling. The RF classifier was trained on exclusively male or female CpG sites, and tested on exclusively male or female CpG sites (denoted by color).

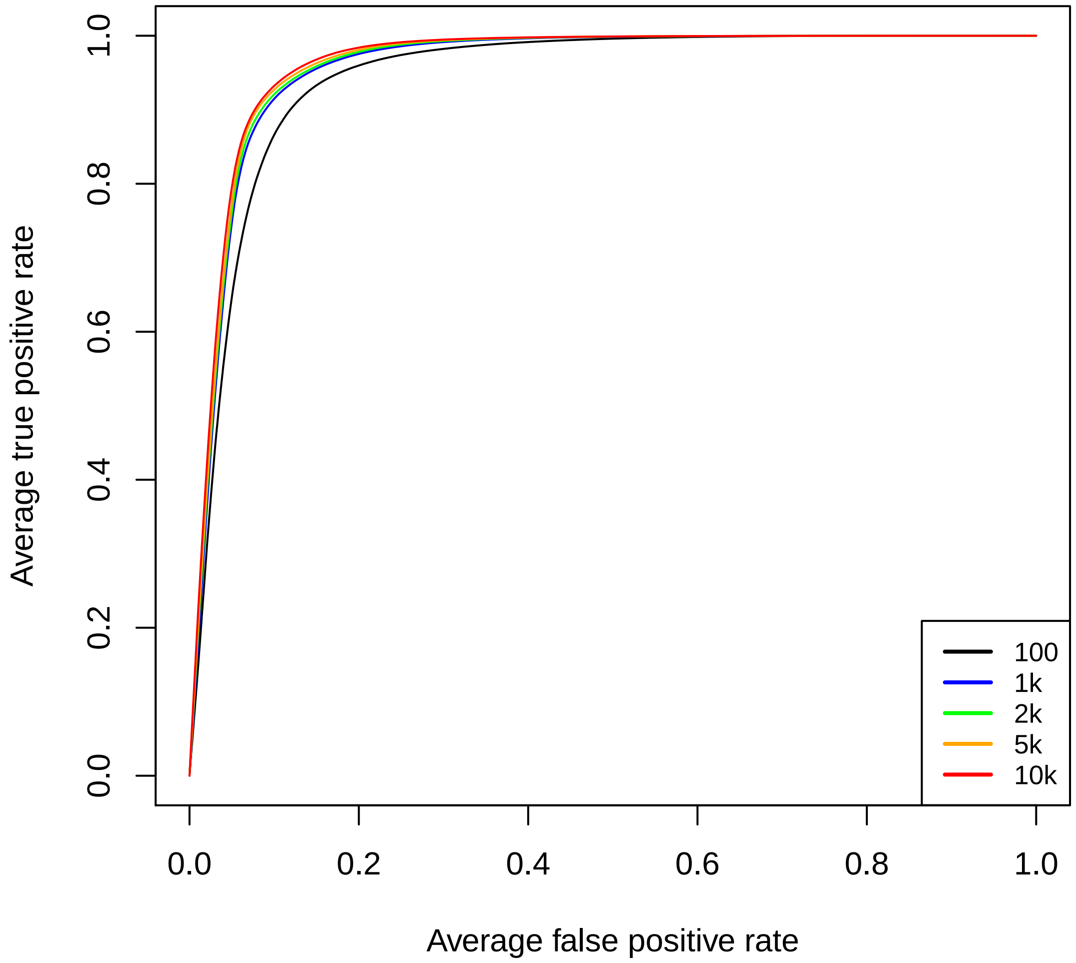

Figure 6: **Generalization error of prediction training size.** Colors represent different training set sizes. For each training set size, the ROC curve is averaged over ten test sets across individuals.

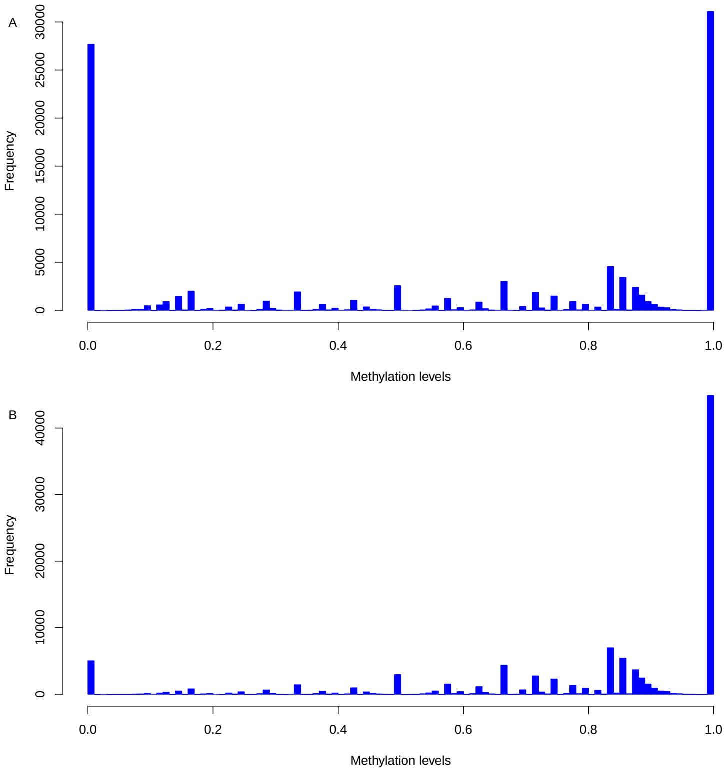

Figure 7: **Distribution of DNA methylation levels at CpG sites from whole-genome bisulfite sequencing data.** Panel A: Distribution of DNA methylation levels across CpG sites from the WGBS data that were categorized as *450K sites*. Panel B: Distribution of DNA methylation levels across CpG sites from the WGBS data that were categorized as *non 450K sites*.

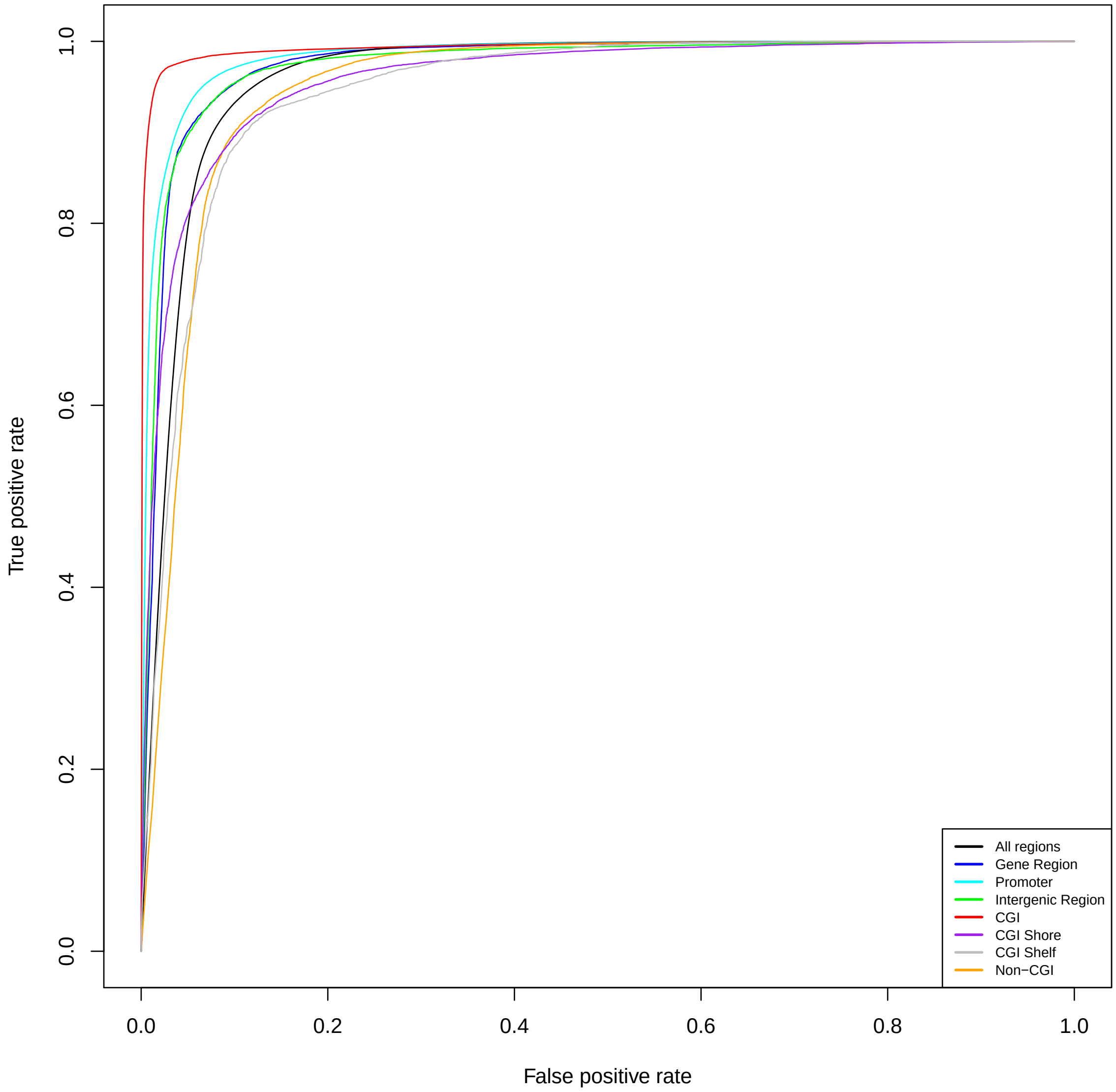

Figure 8: **Prediction performance of region specific methylation status.** ROC curves of region specific methylation status prediction. Colors represent predictions of CpG site methylation status within different genomic regions. For each category, the curve was generated by averaging the results across held out test sets.

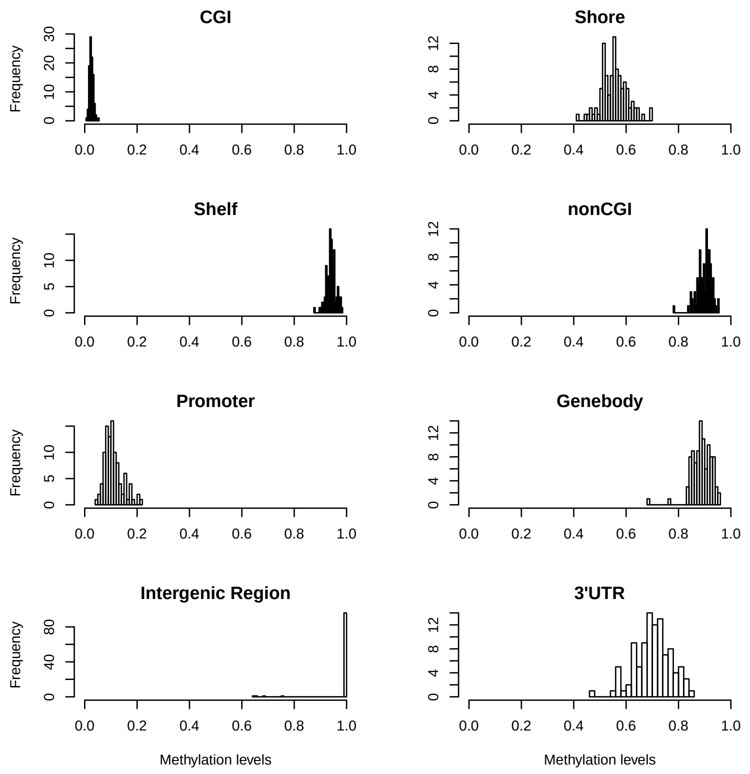

Figure 9: **Histogram of single CpG sites across** 100 **individuals** Histogram of methylation levels at single CpG sites from different genomic regions across 100 individuals.

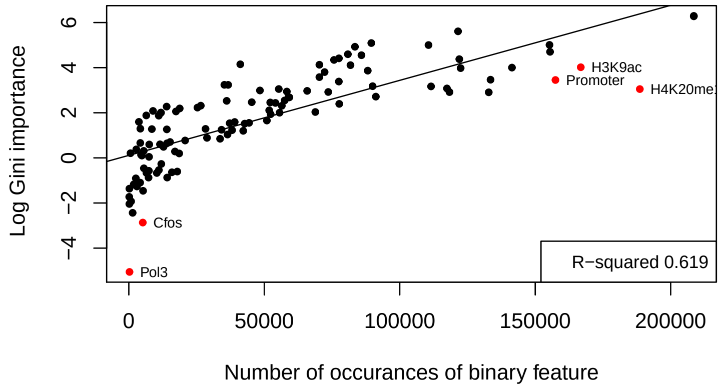

Figure 10: **Correlation of Gini index and the co-occurrence counts of binary features.** The x-axis represents the number of CpG sites co-localized with the corresponding feature (the number of CpG sites that were encoded as '1' for corresponding feature). The y-axis represents the log value of Gini scores of prediction with neighboring CpG site of arbitrary distance. The line was fitted by linear regression. The outliers are highlighted in red. UpMethy: upstream CpG site's methylation status; DownMethy: downstream CpG site's methylation status; Pol3, Cfos, Rxra: TFBSs of Pol3, c-fos and Rxr-α.

References

1. Amoreira C, Hindermann W, Grunau C: **An improved version of the DNA methylation database (MethDB)**. *Nucleic Acids Research* 2003, **31**:75–77, [http://www.nar.oupjournals.org/cgi/doi/10.1093/nar/gkg093].
